# A Physical Perspective to the Inductive Function of Myelin—A Missing Piece of Neuroscience

**DOI:** 10.3389/fncir.2020.562005

**Published:** 2021-01-18

**Authors:** Hao Wang, Jiahui Wang, Guangyi Cai, Yonghong Liu, Yansong Qu, Tianzhun Wu

**Affiliations:** ^1^Institute of Biomedical & Health Engineering, Shenzhen Institutes of Advanced Technology (SIAT), Chinese Academy of Sciences (CAS), Shenzhen, China; ^2^Department of Electrical and Computer Engineering, National University of Singapore, Singapore, Singapore; ^3^Key Laboratory of Health Bioinformatics, Chinese Academy of Sciences, Shenzhen, China

**Keywords:** myelin, opposite spiraling, piezoelectric cell membrane, magnetic resonance imaging, magnetic nerve stimulation

## Abstract

Starting from the inductance in neurons, two physical origins are discussed, which are the coil inductance of myelin and the piezoelectric effect of the cell membrane. The direct evidence of the coil inductance of myelin is the opposite spiraling phenomenon between adjacent myelin sheaths confirmed by previous studies. As for the piezoelectric effect of the cell membrane, which has been well-known in physics, the direct evidence is the mechanical wave accompany with action potential. Therefore, a more complete physical nature of neural signals is provided. In conventional neuroscience, the neural signal is a pure electrical signal. In our new theory, the neural signal is an energy pulse containing electrical, magnetic, and mechanical components. Such a physical understanding of the neural signal and neural systems significantly improve the knowledge of the neurons. On the one hand, we achieve a corrected neural circuit of an inductor-capacitor-capacitor (LCC) form, whose frequency response and electrical characteristics have been validated by previous studies and the modeling fitting of artifacts in our experiments. On the other hand, a number of phenomena observed in neural experiments are explained. In particular, they are the mechanism of magnetic nerve stimulations and ultrasound nerve stimulations, the MRI image contrast issue and Anode Break Excitation. At last, the biological function of myelin is summarized. It is to provide inductance in the process of neural signal, which can enhance the signal speed in peripheral nervous systems and provide frequency modulation function in central nervous systems.

## Introduction

Myelin is a lipid-rich substance that surrounds nerve cell axons. Conventionally, it is often compared to electrical insulation on nerve fibers, inhibiting the ionic current on internodes (Bean, 2007). Thus, the action potential can only be activated on nodes of Ranvier, which are unmyelinated gaps between myelin sheaths. Action potentials traveling down the axon “jump” from node to node, resulting in faster conduction of the action potential. The length of the node of Ranvier is very short, about 1 μm, in peripheral nervous systems (PNS), supporting this explanation of myelin's biological function (Dun, 1970). However, in a recent study of pyramidal neurons in the neocortex, a distinct longitudinal distribution of myelin along individual neurons was observed. Neurons in superficial layers can have long unmyelinated tracts between two myelin sheaths, which is different from the regular myelin profile in PNS (Tomassy et al., 2014). This finding challenges the understanding of myelin as insulating layers, indicating a new concept and mechanism about how information is transmitted and integrated in the brain (Fields, 2014).

In this perspective article, a new physical perspective to understand the biological function of myelin is proposed: the primary role of the myelin is to provide inductance in neuron systems. This inductance plays a significant role in the generation and propagation of neural signals and also induces various unique phenomena in all kinds of neural studies. A comprehensive theory about the inductive function of myelin is illustrated in this study. Finally, the distinct myelin profile in the cortex becomes an inevitable deduction from this theory, revealing its biological importance.

**Scheme 1:**
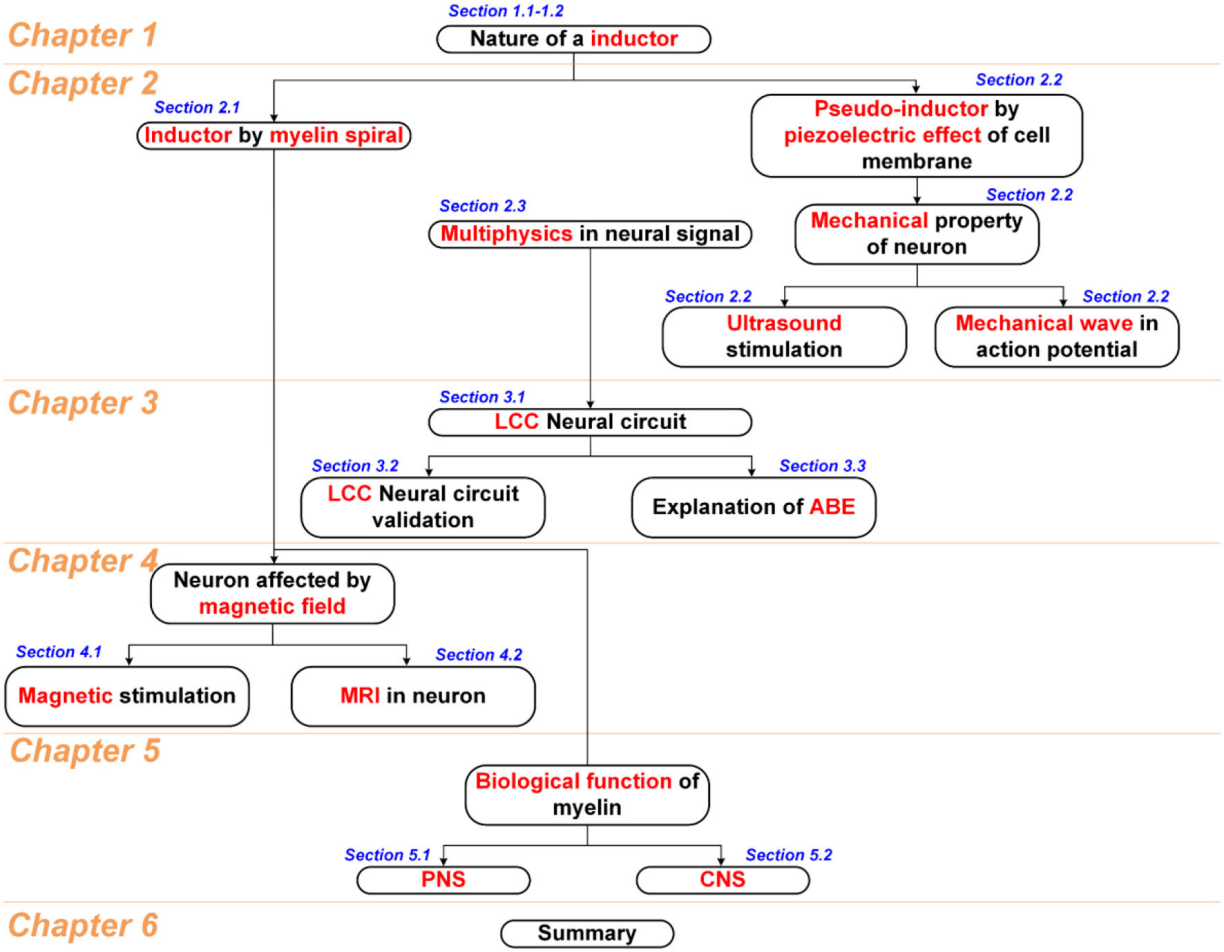
The logic structure of all chapters and sections.

Considering that too many topics of different areas in this study, including fundamental physics, neural circuit, circuit simulation, physiological study, and some biological conjectures, a figure showing the logical connection between all chapters and sections is shown in Scheme 1. Generally, this study starts with an understanding of the nature of the inductance in chapter 1. Then two physical entities are proposed in chapter 2, the inductance generated by the myelin spiral and the equivalent/pseudo inductance by the piezoelectric effect of the cell membrane, to account for the huge inductance observed in the physiological study of neurons. Then based on these two physics, a multiphysics perspective of the neural signal is proposed. In chapter 3, we will have a more detailed discussion about the basic configuration of the neural signal. According to the multiphysics perspective in chapter 2, a new neural circuit of LCC configuration is proposed and validated. Since the myelin spiral acts as a coil inductor to generate an inductance, all the magnetism related phenomena can be explained by the interaction between the myelin spiral and the magnetic field. So in chapter 4, we systematically explained the phenomena in magnetic nerve stimulation and magnetic resonance imaging (MRI). Since the spiraling structure of the myelin can generate inductance, which is completely different from the conventional theory, the biological function of the myelin in nervous systems may be re-illustrated. So in chapter 5, based on the theory developed in this study, new conjectures of biological functions of myelin in the peripheral nervous system and central nervous system are proposed. In the final chapter 6, all phenomena explained in this study is summarized in a figure with logical connections. This figure, together with Scheme 1, builds the framework of the whole theory in this study.

## Chapter 1. How to Understand the Inductance?

The primary purpose of this section is to provide an in-depth understanding of the nature of inductance to biological researchers, who typically do not have a substantial physical background. It will be beneficial for the illustration and understanding of the whole theory proposed in this study.

### The Nature of an Inductor

In an actual circuit, an inductor is an electronic component for storing energy in the form of a magnetic field. However, in most cases, biological researchers are not studying an actual circuit but an equivalent circuit that is modeled from some biological tissue or organism, for instance, an equivalent neural circuit. In this kind of equivalent circuit, an inductor is not an actual unit but a symbol for reproducing the voltage oscillation and resonance frequency measured in electrophysiological tests. Since the voltage oscillation and resonance frequency are the typical characteristics of an RLC circuit, adding an inductor in the equivalent circuit becomes inevitable.

**Figure 1:**
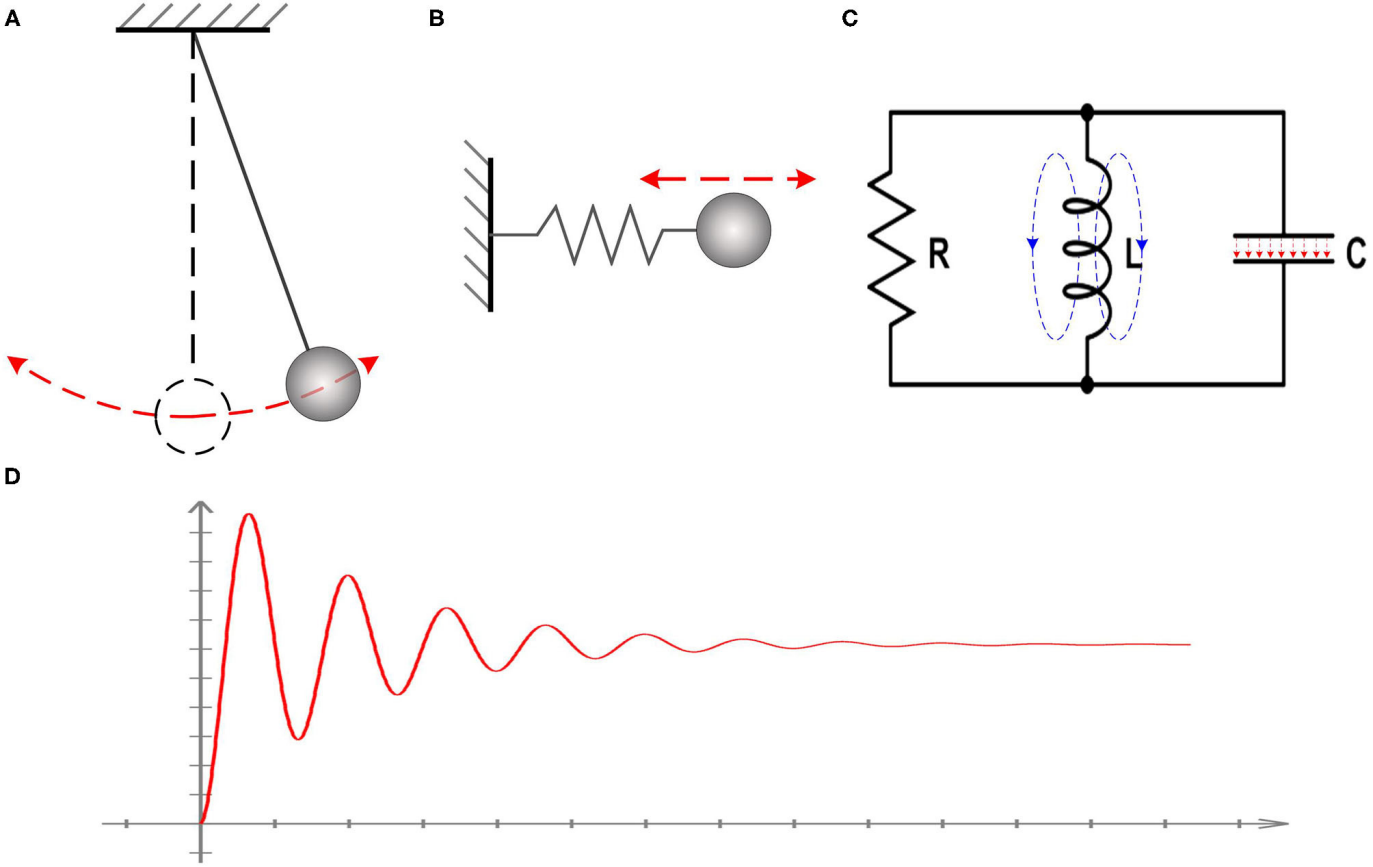
Illustration of the nature of inductance. **(A)** The case of a simple pendulum; **(B)** The case of the one-dimensional harmonic oscillator; **(C)** A system with simple harmonic motion can always be modeled as an RLC circuit; **(D)** The observed voltage oscillation and resonance frequency in all systems with simple harmonic motion.

However, the actual phenomena to be observed in tests are the voltage oscillation and resonance frequency, which is not directly associated with the existence of inductance in the equivalent circuit. There are a lot of cases which can generate oscillation and resonance frequency without the presence of inductance. One example is a simple pendulum, as shown in Figure 1A. Another example is the one-dimensional harmonic oscillator, as shown in Figure 1B. In these two cases, there is no presence of inductor, but the oscillation and resonance frequency exists. A system with simple harmonic motion can always be modeled as an RLC circuit, as shown in Figure 1C. Here we need to emphasize two points, which are critical to the theory in this study:

- The reason for the oscillation and resonance frequency is that the total energy of the whole system has a conversion between different energy forms. In the case of the simple pendulum, the energy conversion is between the gravitational potential energy and kinetic energy. In the case of the one-dimensional harmonic oscillator, the energy conversion is between the elastic potential energy of the spring and kinetic energy of the oscillator. In the case of an actual RLC circuit, the energy conversion is between the electric field in the capacitor and the magnetic field in the coil inductor. Therefore, the inductor in an equivalent circuit means there is an energy conversion between two forms.
- Adding an inductor in the equivalent circuit is to reproduce the oscillation and resonance frequency, as shown in Figure 1D. The inductor itself does not necessarily have a physical meaning, and its value can be unrealistic compared with the one in an actual circuit. In the case of the pendulum, the swinging frequency can be very low, which is about 1 Hz. Based on the equation to calculate the resonance frequency, $f=\frac{1}{2\pi \sqrt{LC}}$, a huge value of the inductance can be obtained. This huge inductance can never happen in an actual circuit but is quite normal in an equivalent circuit.

### The Potential Fallacy of Conventional Neuroscience

By understanding the two points mentioned above, it is quite clear to see what is theoretically inadequate with the conventional neuroscience. There is a large inductance in neural systems, which has been reported in a lot of studies (Cole and Baker, 1941; Curtis and Cole, 1942; Hodgkin and Huxley, 1952; Sjodin and Mullins, 1958; Araki et al., 1961; Freeman, 1961; Huxley, 1963; Ranck, 1963; Guttman, 1969; Mauro et al., 1970; Scott, 1971; Homblé and Jenard, 1984; Hutcheon and Yarom, 2000; Dwyer et al., 2012; Thomas, 2013; Mosgaard et al., 2015; Kumai, 2017; Rossi and Griffith, 2017). The evidence of this inductance, as explained above, is the voltage oscillation and resonance frequency measured in experiments. The first study of this large inductance is the paper proposing the H-H model (Hodgkin and Huxley, 1952). The measured inductance can be about 0.21 to 0.39 H, which is much higher than a reasonable value of a physical coil inductor. Based on their proposal, this large inductance is induced by the impedance change of the ion channels. This bizarre phenomenon also aroused lots of other theoretical guesses in later studies, such as frequency-dependent membrane capacitance (Howell et al., 2015), negative resistance (Rissman, 1977), and negative capacitance (Takashima and Schwan, 1974).

Nevertheless, all of them were misled by two points emphasized here:

- They considered the voltage oscillation and resonance frequency as the evidence of the inductance.
- They believed that a coil is an exclusive origin accounting for the inductance in an equivalent circuit.

With the clarification of these two points, a better theoretical hypothesis can be taken into consideration. The inductance in the neural circuit means there is a kind of biological structure that can store the energy in a non-electrical form. Since the cell membrane is typically modeled as a capacitor, the energy conversion happens between the electrical field stored in the cell membrane and some unknown form stored in an unknown biological structure.

The same idea was first proposed by Cole (1941). It was said in the paper that the measured large inductance in neurons could be raised from the piezoelectric effect of the cell membrane. The energy conversion happens between the electrical field in the cell membrane and the surface tension by the piezoelectric effect. Since the inductance is calculated from the resonance frequency $f=\frac{1}{2\pi \sqrt{LC}}$, its value can be quite large if the resonance frequency is very low. However, at the time of 1941, the lipid bilayer structure of the cell membrane, which is naturally piezoelectric, remains unknown to Kenneth S. Cole, he proposed this idea as a hypothesis. It is a pity that his opinion drew no attention in later research. We will make a detailed discussion of this point below.

## Chapter 2. How Does the Myelin Generate the Inductance?

In the following section, we will propose some physical theories to explain how myelin generates inductance. The scientific paradigm for the validation of theories is as follow:

- Predict/deduce/explain a unique experimental phenomenon from the theory, which cannot be well-explained in conventional theories.
- Validate the phenomenon experimentally by either our researches or others' studies.
- If the predicted/deduced phenomenon is validated/observed in experiments, then the theory is validated.

This is a standard scientific paradigm, which widely applied for all kinds of theory validation, such as Newton's law of universal gravitation (by predicting/explain the elliptical orbits of planets) and the theory of relativity (by predicting the angle of light deflection by the sun). There are lots of phenomena/predictions proposed in this theory. All of them are summarized in **Figure 14**.

### Myelin Can Act as a Coil Inductor

**Figure 2:**
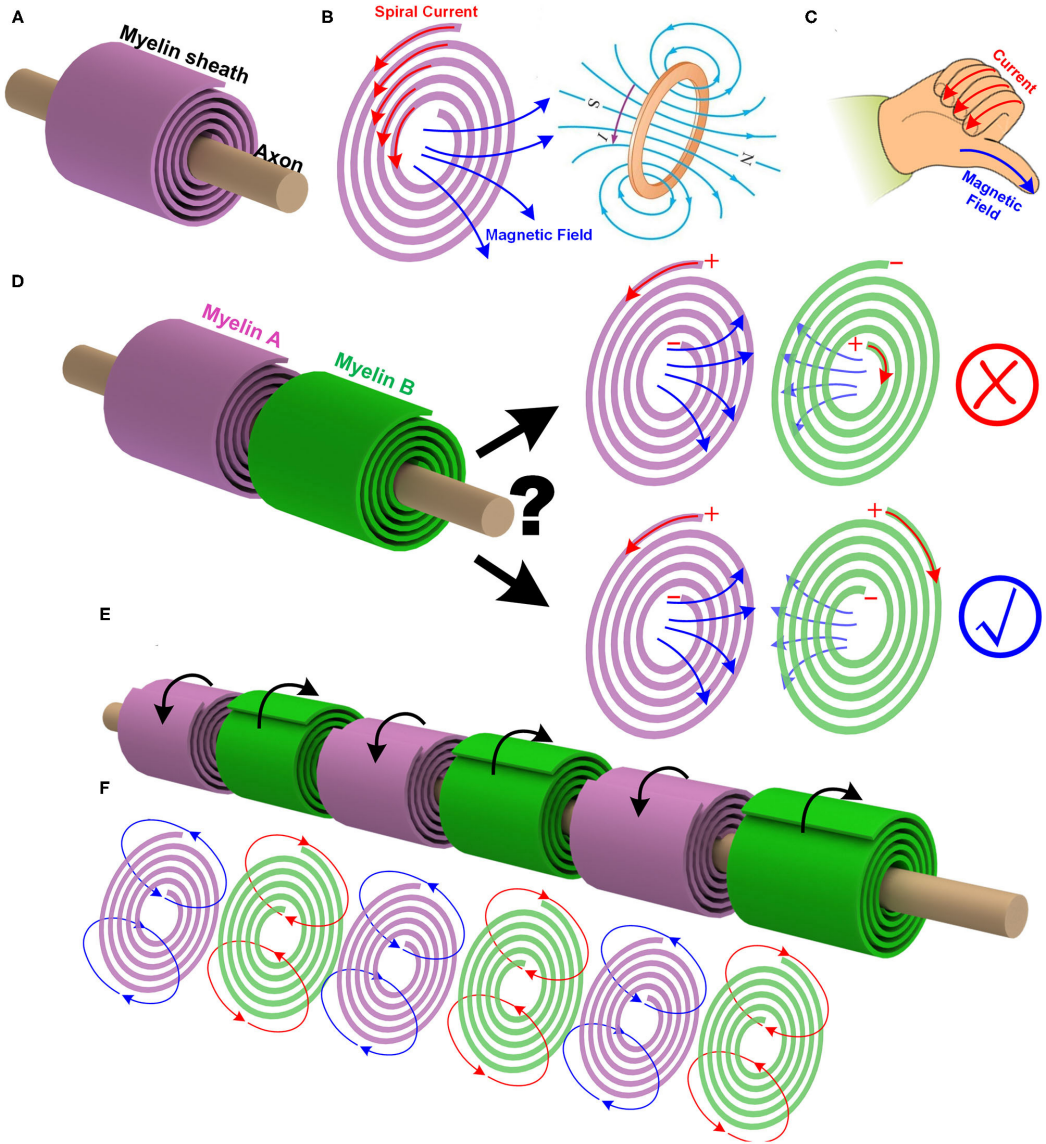
The myelin can act as a coil inductor. **(A)** The myelin wrapping around the axon as a coil; **(B)** The spiral current inside the myelin generates a magnetic field; **(C)** The right-hand screw rule to determine the direction of the magnetic field generated by a coil; **(D)** The mutual inductance between adjacent myelin sheaths and the effect of the spiraling directions; **(E)** The adjacent myelin sheaths always have opposite spiraling directions; **(F)** The magnetic field generated by each myelin sheath will be opposite to each other.

The myelin sheath wrapping around the axon is quite similar to a coil, as shown in Figure 2A. When an electric field is applied between the outside-terminal and the inside-terminal, the moving ions inside the myelin will generate a spiral current, as shown in Figure 2B. Just like the current in a coil, this spiral current produces a magnetic field whose direction can be determined by the right-hand screw rule, as shown in Figure 2C. So a myelin sheath acts as a coil inductor to generate a magnetic field.

To validate the existence of this coil inductor, we need to make a unique experimental prediction, which is from a reasoning process as below:

- In peripheral nervous systems, the adjacent myelin sheaths are very close to each other. If the myelin sheath acts as a coil inductor, there will be a mutual inductance. It means the magnetic field generated by one myelin sheath can induce another magnetic field on the next myelin sheath, as shown in Figure 2D.
- This magnetic generated by the myelin sheath is also part of the neural signal. So it contains the information to be delivered by the neural signal.
- At one instant, i.e., at the depolarization phase of the action potential, the voltage polarity on myelin sheath A is positive outside and negative inside. Then the current inside the myelin A is anti-clockwise, and the right-hand screw rule determines the direction of the magnetic field. Based on the Lenz's law, the direction of the induced magnetic field of myelin B is opposite to that of the myelin A. Again, based on the right-hand screw rule, the direction of the current inside myelin B is clockwise, as shown in Figure 2D.
- The spiraling direction of myelin B determines its voltage polarity.
- A critical question is: Should the voltage polarities on myelin A and B be the same or opposite, as shown in Figure 2D?
- Since this magnetic field is part of the neural signal, the information to be delivered from myelin A to myelin B should be the voltage polarity. So myelin A and myelin B should share the same voltage polarity, which is positive outside and negative inside. Based on the right-hand screw rule, the spiraling direction of myelin B, from outside to inside, is clockwise, which is opposite to that of myelin A.

Thus, a unique experimental phenomenon is predicted. In peripheral nervous systems, where the node of Ranvier is always very short, the spiraling directions of the adjacent myelin sheaths are always opposite to each other, as shown in Figure 2E (P1 in **Figure 14**). Currently, no other theories or models can give this prediction. Figure 2 shows this intuitive reasoning process. General speaking, the adjacent myelin sheaths should have a positive mutual inductance (opposite spiraling). This positive mutual inductance will be beneficial for the neural signal propagation. A more quantitative validation process by circuit simulation will be provided in **Figure 12** to explain this beneficial effect.

This phenomenon has been confirmed in other studies (Uzman and Nogueira-Graf, 1957; Bunge et al., 1989; Armati and Mathey, 2013), and the conclusion is quite clear. The earliest report of this phenomenon is in a paper on the Scanning electron microscope (SEM) observation of the biological structure of myelin sheaths and node of Ranvier in mouse sciatic nerve, published in 1957 (Uzman and Nogueira-Graf, 1957). The original sentence is quoted here:

*At the junction of two Schwann cells along an axon, the directions of the lamellar overhang of the myelin endings are of opposite sense*.

Another review on the biological function of myelin, published in 2013 (Armati and Mathey, 2013), made a more unequivocal statement, quoted here:

*Of unknown significance was the observation by Van Geren who first described the spiralling of the Schwann cell, that each Schwann cell spiral is in the opposite direction to its neighbour*.

Thus, the currently experimental observation does support our theory: the coil structure of the myelin sheath can store energy in the form of a magnetic field.

There is another interesting prediction from this theory. Due to the Lenz's law and the opposite spiraling phenomenon, the magnetic field generated by each myelin sheath will be opposite to each other, as shown in Figure 2F. Generally, they all cancel with each other, making the external measurement of this magnetic field very difficult. Thus, the measured magnetic field in the neural signal is negligible (Roth and Wikswo, 1985) (P2 in **Figure 14**). In other words, although the magnetic field does exist in the action potential, it may not be so measurable. The best evidence for the existence of this magnetic field is the opposite spiraling phenomenon.

Meanwhile, it is emphasized here that the experimental setup used for the measurement of the magnetic field in neural signals in a previous study (Roth and Wikswo, 1985) cannot be applied for the measurement of the magnetic field generated by the myelin sheaths. Previously, it was assumed that it is the current along the axon to generate the magnetic field, which has a circular direction around the axon. However, in our theory, the spiraling current along the myelin wrapping sheaths generates the magnetic field, whose direction is entirely different. Therefore, it requires a different experimental setup for the measurement, which is detailed explained in the Supplementary S4.

**Figure 3:**
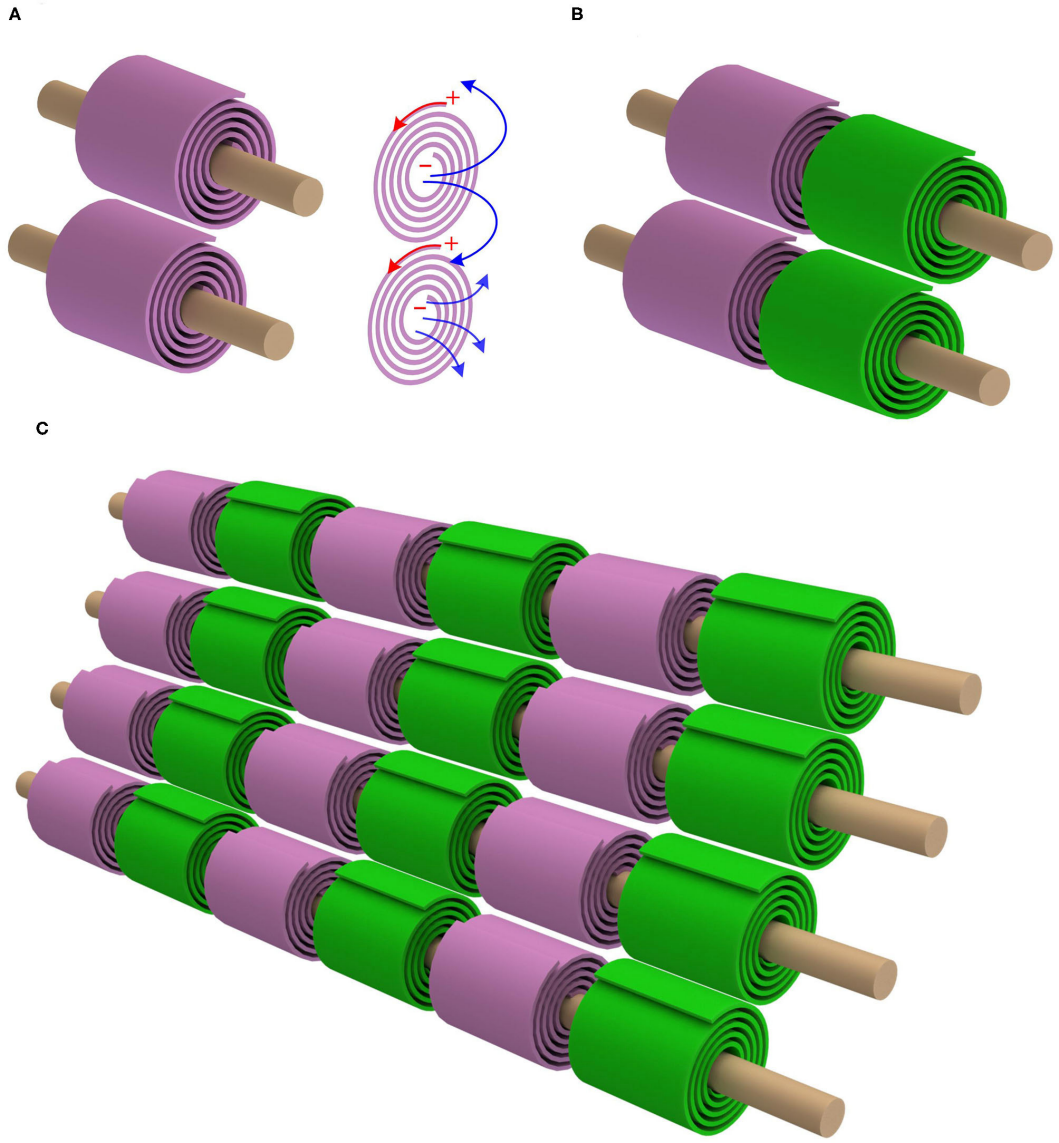
The mutual inductance between adjacent axons. **(A)** The myelin sheaths on adjacent axons shall have the same spiraling direction to achieve a positive mutual inductance; **(B)** By accounting for the opposite spiraling effect between adjacent myelin sheaths on the same axon, the internodes and Ranvier nodes should be aligned; **(C)** The alignment effect for multiple myelinated axons.

As seen, for achieving a positive mutual inductance, the adjacent myelin sheaths' spiraling directions should be the opposite. Let's take one step further. The mutual inductance also exists between the myelin sheaths on adjacent axons. Based on the same principle of the positive mutual inductance, the myelin sheaths on the adjacent axons should have the same spiraling direction, as shown in Figure 3A (P23 in **Figure 14**). This is also a reported phenomenon, quoted here (Richards et al., 1983):

*An analysis of the direction of myelination of fibers in the optic tract of kittens shows that the direction of wrapping of neighboring fibers is not random. Adjacent fibers in contact with the same glial process tend to be wrapped in the same direction*.

Moreover, we can further predict in which condition this same spiraling phenomenon shall occur or disappear. Since the mutual inductance exists only when the myelin sheaths are close to each other, this same spiraling phenomenon only happens when the neural fibers are compact (Richards et al., 1983). If the neural fibers are distributed sparsely, the spiraling directions of the myelin sheaths on different axons shall be random, which is also reported, quoted here (71):

*Moreover, our finding that the clockwise - counterclockwise course of the myelin spiral varies randomly within one unit, contradicts the ‘model' of myelination formulated by Richards et al. (**1983**)*.

Then we get an remarkable prediction, which is shown in Figures 3B,C). Since the adjacent myelin sheaths on the same axon have the opposite spiraling directions and the myelin sheaths on adjacent axons have the same spiraling direction, the internodes and Ranvier nodes should inevitably have a good alignment. Therefore, when the nerve fibers are compact in PNS, internodes and Ranviers nodes of these nerve fibers should be aligned. This is a unique prediction of our theory, which cannot be derived from any other theories/models. The validation of this phenomenon can substantially support our theory.

However, this inductance from the spiraling of the myelin sheaths cannot fully explain the large inductance in neural systems:

- This large inductance was firstly measured on the giant squid axon, which is an unmyelinated nerve. So this inductance is not associated with the myelin.
- Considering the dimension and the coil turns of the myelin sheath, the generated inductance can only be a small value, which is far less than the measured large inductance.

Since the myelin is a sheet wrapping around the axon, whose inductance cannot be directly calculated from any empirical equations, a simulation by COMSOL is provided in Supplementary S3 to evaluate the inductance generated by the myelin sheath. The value of the inductance will increase quadratically with the number of layers and decrease with the length of myelin sheath. Based on the simulation, a myelin sheath with 150 layers, its inductance should be nH range.

Therefore, the origin of this large inductance, just as Kenneth S. Cole guessed in 1941, is an equivalent inductance generated by the piezoelectric effect of the cell membrane.

### The Equivalent Inductance Generated by the Piezoelectric Effect of the Cell Membrane

The piezoelectric effect of the cell membrane was first proposed by Cole (1941) to explain the large inductance measured in the giant squid axon. The energy conversion between the electric field and the surface tension is the origin of the large inductance measured. The original statement by Kenneth S. Cole is quoted here:

*There is another and more common class of inductances arising from mechanical motions, and the most familiar example of these is the piezoelectric crystal, such as quartz or Rochelle salt*.

*It may seem quite unreasonable to suppose that the axon membrane may be piezoelectric with a natural frequency of a few hundred cycles, but in the present state of our information this possibility cannot be excluded*.

*In this discussion of the possible sources of inductance it has been emphasized that the common association of an inductance with a magnetic field may be misleading*.

Now the question is whether the cell membrane has a piezoelectric effect. Actually, it does. However, instead of the piezoelectric effect, the flexoelectric effect is a more precise definition for the property of the cell membrane. The definition of flexoelectric effect is quoted here (Tagantsev, 1986, 1991; Petrov, 2006; Yudin and Tagantsev, 2013;Zubko et al., 2013):

*Flexoelectricity is a property of a dielectric material whereby it exhibits a spontaneous electrical polarization induced by a strain gradient. Flexoelectricity is closely related to piezoelectricity, but while piezoelectricity refers to polarization due to uniform strain, flexoelectricity refers specifically to polarization due to strain that changes from point to point in the material. This nonuniform strain breaks centrosymmetry, meaning that unlike in piezoelectricity, flexoelectric effects can occur in centrosymmetric crystal structures*.

**Figure 4:**
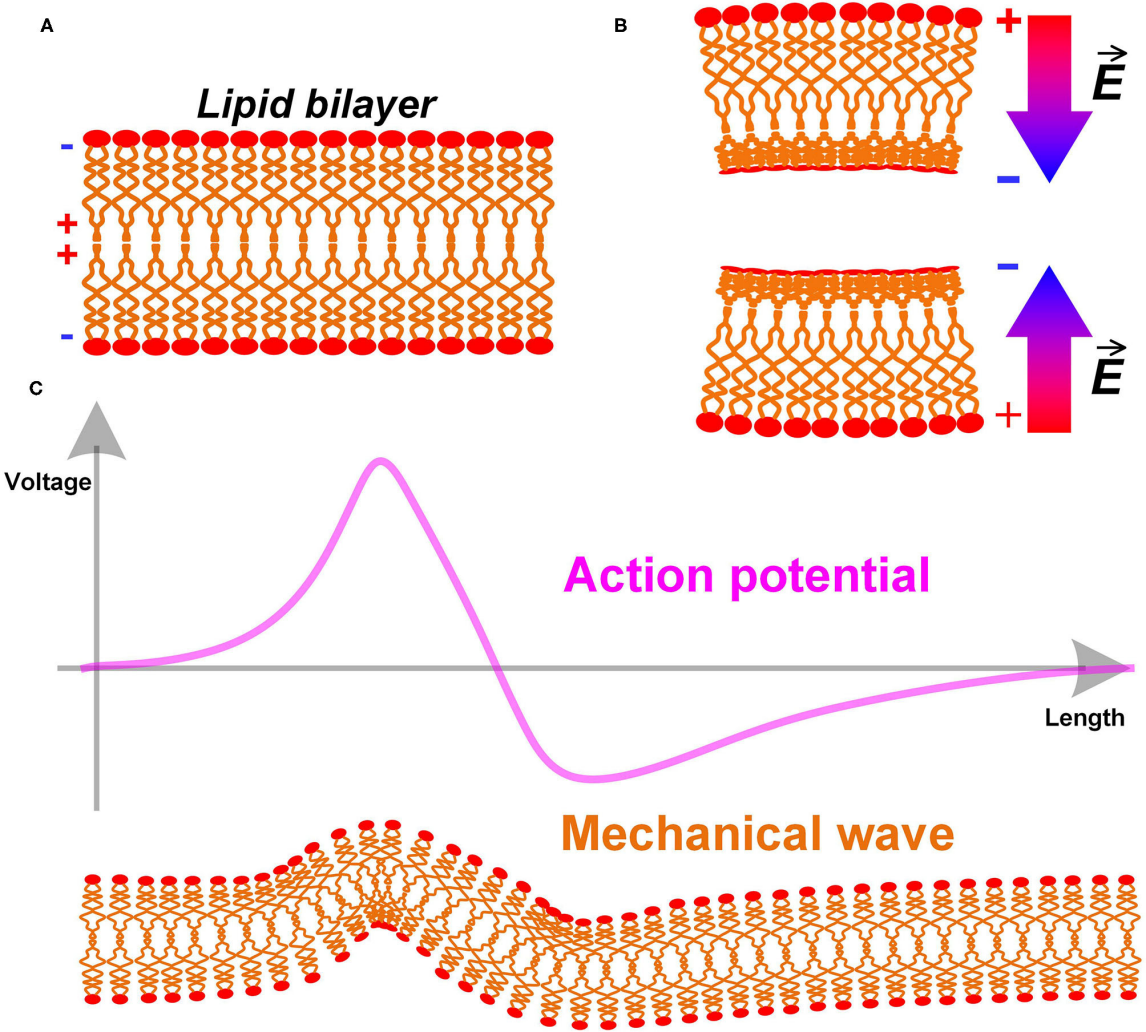
**(A)** The lipid bilayer structure of cell membrane; **(B)** The bending of the cell membrane breaks the centrosymmetry and generates an extra electric field; **(C)** The action potential as an electric field will induce a mechanical wave with it.

Here is a simple analysis of the biological structure of the cell membrane. The cell membrane, also named as the plasma membrane, has a lipid bilayer structure, as shown in Figure 4A. The lipid molecules are dipoles, with positive tails toward the center and negative tails toward the extra- and intracellular fluid (shown as the + and – signs in Figure 4A) (Andersen and Koeppe, 2007). Apparently, this is a centrosymmetric structure. When the cell membrane is bend, its centrosymmetry is broken, resulting in a non-uniform charge re-distribution. An extra electric field/voltage is thus generated, as shown in Figure 4B. This is entirely consistent with the definition of the flexoelectric effect quoted above. Therefore, the cell membrane has a flexoelectric effect, which is a kind of special piezoelectric effect. We can consider the cell membrane as a piezoelectric membrane, which has already been a common sense in biophysics. A. K. Tagantsev and Alexander G. Petrov have published many papers on flexoelectricity (Zubko et al., 2013). The flexoelectric effect of the lipid bilayer and its biological effect is well-studied and summarized in the review by Petrov (2006).

A direct prediction is a mechanical wave accompany by the action potential (P3 in **Figure 14**). Since the cell membrane is piezoelectric, it will deform by applying an electric field. The action potential, which is an electric field, can generate a deformation of the cell membrane, as shown in Figure 4C. In terms of measurement, this deformation will be a moving mechanical vibration, which is a mechanical wave. This is a common phenomenon in piezoelectric membranes. A one-step further prediction is that the mechanical wave has the same speed as the action potential. Due to the piezoelectric effect, the mechanical wave and the action potential are coupled with each other. In other words, this mechanical wave is part of the neural signal.

The measurement of this mechanical wave has been completed by Thomas Heimburg's group (Gonzalez-Perez et al., 2016). The mechanical wave was measured on the lobster neuron, which has the same speed as the action potential (Appali et al., 2012). A soliton theory is thus built, as an alternative theory to the H-H model, to explain and calculate the speed of the action potential from a purely mechanical perspective. However, if we do not try calculating the propagation speed, a qualitative conclusion that the mechanical wave has the same speed as the action potential can be obtained without building a calculational model. Some neuroscientists may care more about whether we can build a more completed calculational model. We will make a detailed discussion about this question in the next section.

Another deduction is the mechanism of ultrasound nerve stimulation (P4 in **Figure 14**). The activation of an action potential requires an electric field upon the cell membrane, which can be applied by neural electrodes in electrical nerve stimulations. Due to the piezoelectric effect of the cell membrane, now this electric field can be generated by applying a surface deformation. The ultrasound is an effective method to produce a high-frequency deformation by an acoustic wave. A one-step further prediction is that a lower frequency ultrasound has higher efficiency on nerve stimulations (P5 in **Figure 14**). A piezoelectric membrane can have a higher vibration amplitude when the external stimulus is closer to its resonance frequency. Compared with the resonance frequency of the cell membrane, measured in kHz level or even lower (Evans, 1972; Li and Bak, 1976; Hartmann et al., 1984; Kral et al., 1998), the frequency of the ultrasound is much higher, ranging from 500 kHz to several MHz. So the energy absorption efficiency by the cell membrane is very low. Under these circumstances, reducing the ultrasound frequency means getting closer to the resonance frequency of the cell membrane. This will surely increase the energy absorption efficiency by the cell membrane, resulting in higher efficiency of nerve stimulations.

The actual experimental observation is strictly consistent with the above prediction. The ultrasound with a lower frequency can achieve a higher stimulation efficiency, quoted here (Ye et al., 2016):

*We did find a clear trend of reduced efficacy as the frequency increased, showing that increased spatial peak intensities were required to achieve the same success rates compared to lower frequencies*.

It is noted that a similar mechanical wave hypothesis was also proposed by Rvachev's study (Rvachev, 2010), in which the physical origin of this mechanical wave is assumed to be the filament contraction induced by the Ca^2+^ ion flux. Based on a purely mechanical perspective, the propagation speed and its effect on the activation of ion channels are explained, which are fundamentally different from the theory proposed in this study. However, the proposed explanation of the Meyer–Overton rule (Yamasaki et al., 1899; Overton, 1901), which is about the effectiveness of anesthetics, shows the explanatory power of this mechanical wave and the importance of its rule in the propagation of the neural signal.

### A Renewed Understanding of the Multiphysics in Neural Signal and Myelin

**Figure 5:**
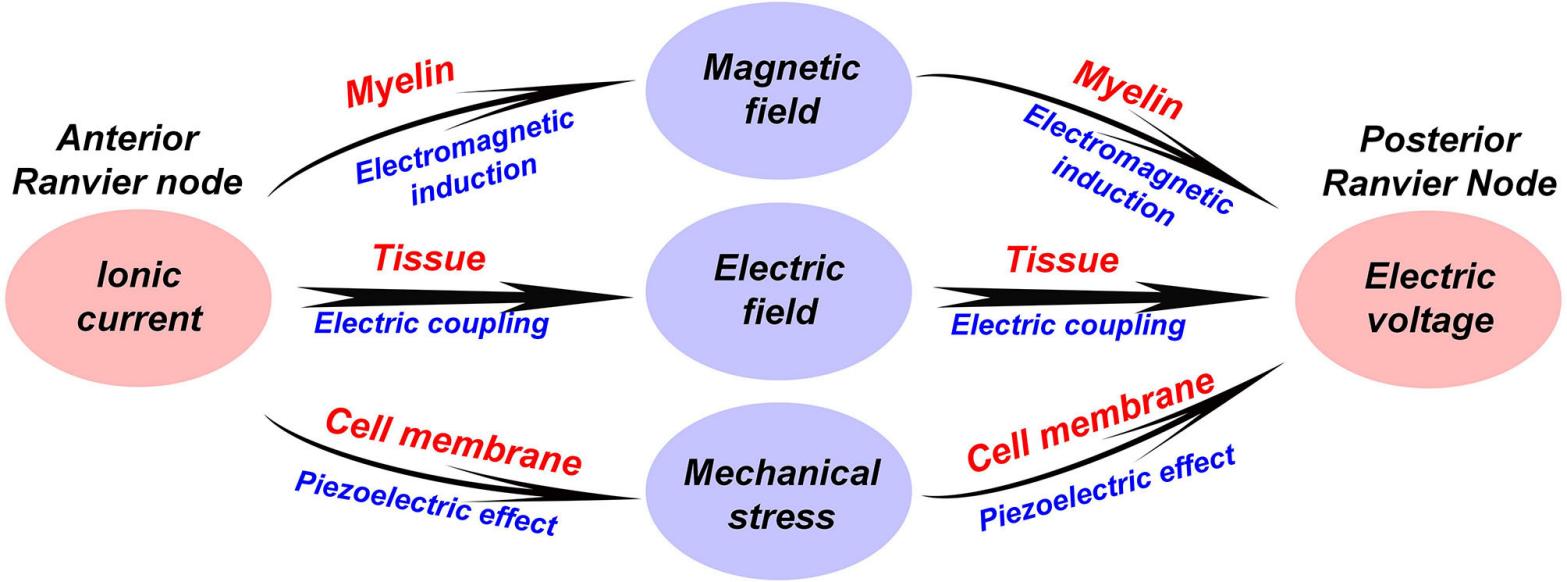
A multi-physical perspective to understand the neural signal and the biological function of the myelin.

In the above sections, we have introduced two new physical mechanisms in the neural signal process, which are electromagnetic induction and piezoelectric effect. Therefore, apart from the electrical signal, the neural signal also consists of a magnetic field and a mechanical wave. Here, a more completed comprehension of the neural signal on myelinated nerves is proposed, as shown in Figure 5:

- A neural signal is an energy pulse containing electrical, magnetic, and mechanical components.
- The propagation of the neural signal is a complex multi-physical process, including electrical field coupling, electromagnetic induction, and piezoelectric effect.
- The function of the node of Ranvier is to replenish energy to this energy pulse, compensating the energy loss during the signal transmission.

For unmyelinated nerves, the multi-physical process will be simpler: the electromagnetic induction process is excluded.

With this completed image, the limitation of the conventional theory is quite obvious. For all models developed from the H-H model, the action potential is considered as a pure electrical signal. In the soliton theory (Appali et al., 2012), which is an alternative theory to the H-H model, the action potential is considered as adiabatic pulse with no energy loss, called solitary wave, during the propagation. This solitary wave is mainly generated by the lipid transition by the cell membrane. Here we proposed another perspective. The neural signal is neither a pure electric signal, nor necessarily an adiabatic pulse. It is almost impossible to make the propagation of the magnetic component without any loss. The neural signal should be a multi-physical process involving electrical, magnetic, and mechanical physics.

Meanwhile, the myelin sheath, referring to both the Schwann cell and oligodendrocyte (Kroepfl et al., 1996; Calderón and DeVries, 1997), also share the same biological structure of the cell membrane as the axon, which is a lipid bilayer and has a piezoelectric effect. Thus, the myelin sheath wrapping around the axon acts as multi-player piezoelectric layers, which also play a role in the propagation of the mechanical wave in the neural signal. Qualitative analysis is as follow:

- Due to the increased number of stacking layers, the piezoelectric effect will be more significant.
- The thickness of the axon is higher, increasing Young's modulus. Its resonance frequency is boosted up, increasing the propagation speed of the mechanical wave. It follows the same mechanism as sound waves travel faster in harder media. This explains how myelin enhances the speed of the neural signal from a mechanical perspective.

As seen, the biological function of the myelin also requires a multi-physical interpretation. Myelin is far more than an insulating layer.

In the next chapter, based on the multiphysics introduced in this section, a corrected neural circuit is proposed for explaining quite a lot of experimental phenomena that confuse people.

## Chapter 3. The Equivalent Circuit of the Neuron

The neural circuit is the basis of the whole neuroscience. Start from the cable theory, all neural models are built based on an RC circuit form. There are so many inductance induced phenomena. To explain them, so many hypotheses are proposed, such as impedance change of the ion channels (Huxley, 1963), virtual-cathode hypothesis (Ranjan et al., 1998), frequency-dependent membrane capacitance (Howell et al., 2015), negative resistance (Rissman, 1977) and negative capacitance (Takashima and Schwan, 1974). The correction of the neural circuit by adding the inductance surely can immediately make everything easily explained, but also induces the collapse of the whole theory system. Therefore, without a reasonable explanation of the physical entity to generate this large inductance, the voice questioning the correctness of the RC circuit form cannot be acknowledged by the mainstream academic community.

In the previous chapter, we already gave a detailed explanation of the entities producing the large inductance in neurons (P6 in **Figure 14**), solving the major issue of adding the inductance in the neural circuit. In this chapter, a corrected neural circuit is derived and further validated by experiments.

### The Basic Configuration of the Neural Circuit

**Figure 6:**
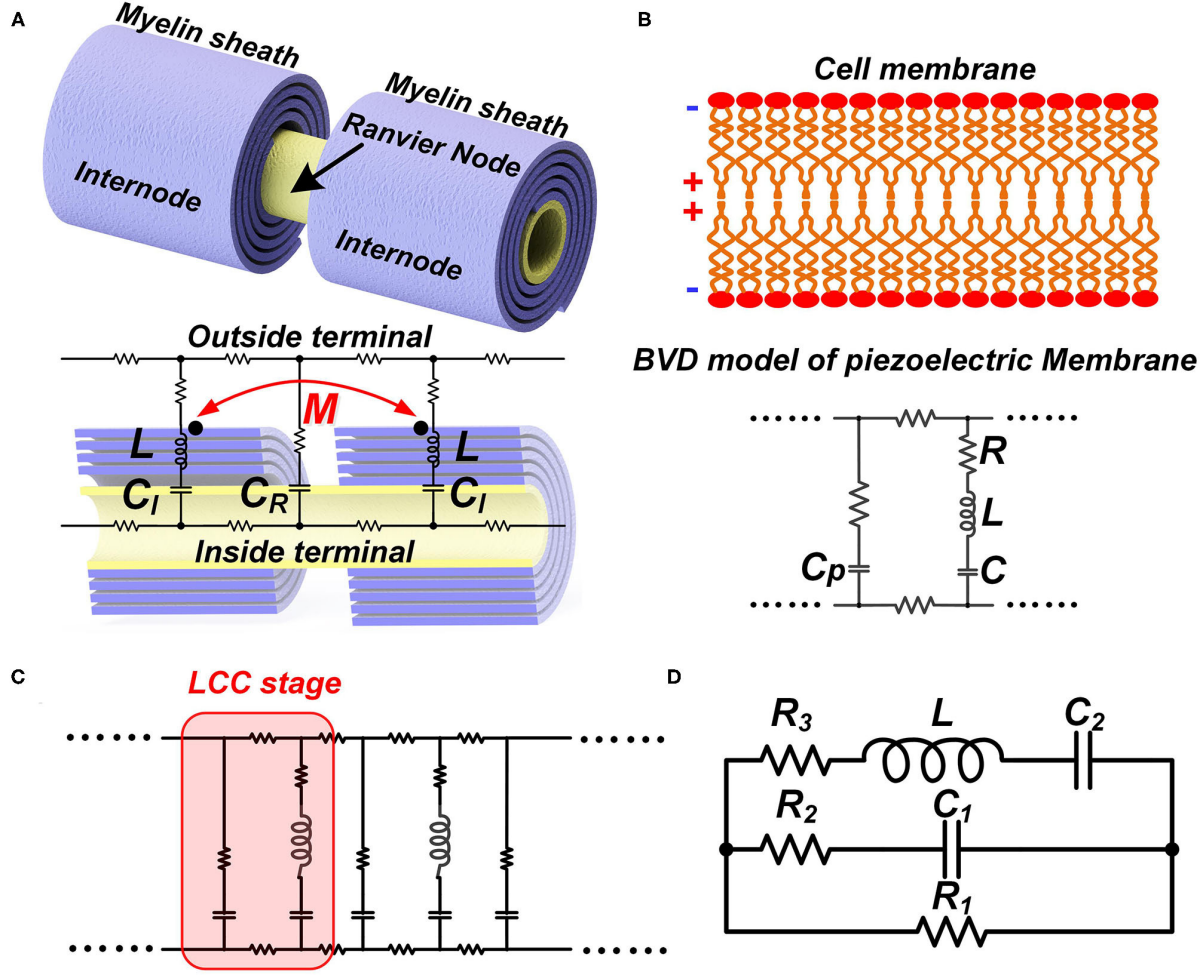
The LCC form of the neural circuit. **(A)** The equivalent circuit of a myelinated nerve by considering the myelin as an inductor; **(B)** The equivalent circuit of a cell membrane by considering its piezoelectric effect; **(C)** The circuit cascade of LCC stages; **(D)** The lumped parameter circuit to simply the cascade circuit.

Like the conventional theory, we build a macroscale neural circuit by modeling the cell membrane as a capacitor. The myelin as a coil is modeled as an inductor. For a myelinated nerve, the part of the internode is modeled as an inductor-capacitor (LC) circuit in series. The node of Ranvier is modeled as a capacitor. The rest parts are modeled as resistors, as shown in Figure 6A. Then the whole myelinated axon is a circuit cascade with each stage as an LC branch connected with a capacitor in parallel, which is called an LCC circuit, as shown in Figure 6C. Due to the mutual inductance between adjacent myelin sheaths, a parameter of mutual inductance is added in the circuit.

Then we build a microscale neural circuit by considering the piezoelectric effect of the cell membrane. Here we can leverage the sophisticated Butterworth-Van Dyke (BVD) model (Larson et al., 2000; Arnau et al., 2001), which is well-established in the study of piezoelectric membranes, as shown in Figure 6B. Here, *C*_*P*_ is the static capacitance of the cell membrane, determined by its area, thickness, and dielectric constant. In the right branch are the motional *C*, *L*, and *R*, representing the propagation of the acoustic waves in the piezoelectric material and defining its acoustic properties. As seen, this is also an LCC circuit. Then the whole axon of both myelinated nerves and unmyelinated nerves can be a cascade of LCC stages, as shown in Figure 6C.

Now we have two neural circuits derived from different levels. In the macroscale circuit, the whole myelin is modeled as an individual inductor. This circuit allows us to assign the mutual inductance. In the microscale circuit, each segment of the cell membrane, including the cell membrane of the myelin sheath, is modeled as an LCC circuit representing the piezoelectric effect. If we want to include the inductance of the myelin spiral in the circuit of each myelin segment, we need to add one inductor, representing the inductance of that segment, in parallel with each LCC circuit. Then one myelin sheath can be modeled as a circuit network, which is a distribute-parameter circuit containing a lot of stages of LCC in parallel with inductors. In this circuit network, the mutual inductance between adjacent myelin sheaths cannot be assigned. If the mutual inductance between adjacent myelin sheaths cannot be assigned, the meaning of considering the myelin spiral as an inductor also disappears. Therefore, a neural circuit to account for all physics involved in the neural signal is not feasible. Thus, a complete multi-physical neuron model cannot be achieved by the modification or correction of the H-H model, whose framework is a neural circuit with differential equations. We need to explore new methods for the development of a global model/theory.

However, the microscale circuit and the macroscale circuit accidentally share the same LCC circuit form. Thus, we can make a bold but reasonable hypothesis that the neural circuit follows an LCC cascade if we only care about the electrical characterization (P7 in **Figure 14**). Meanwhile, the simplified lumped-parameter circuit of a complex neuron network, whose basic element is an LCC stage, should also be an LCC form, as shown in Figure 6D. As a partial model/theory, this neural circuit is still quite useful for the electrical characterization and phenomena explanations in neuroscience. We will demonstrate how to use this circuit in the following sections.

### The Validation of the Neural Circuit of LCC Form

#### The Validation of Frequency Response

**Figure 7:**
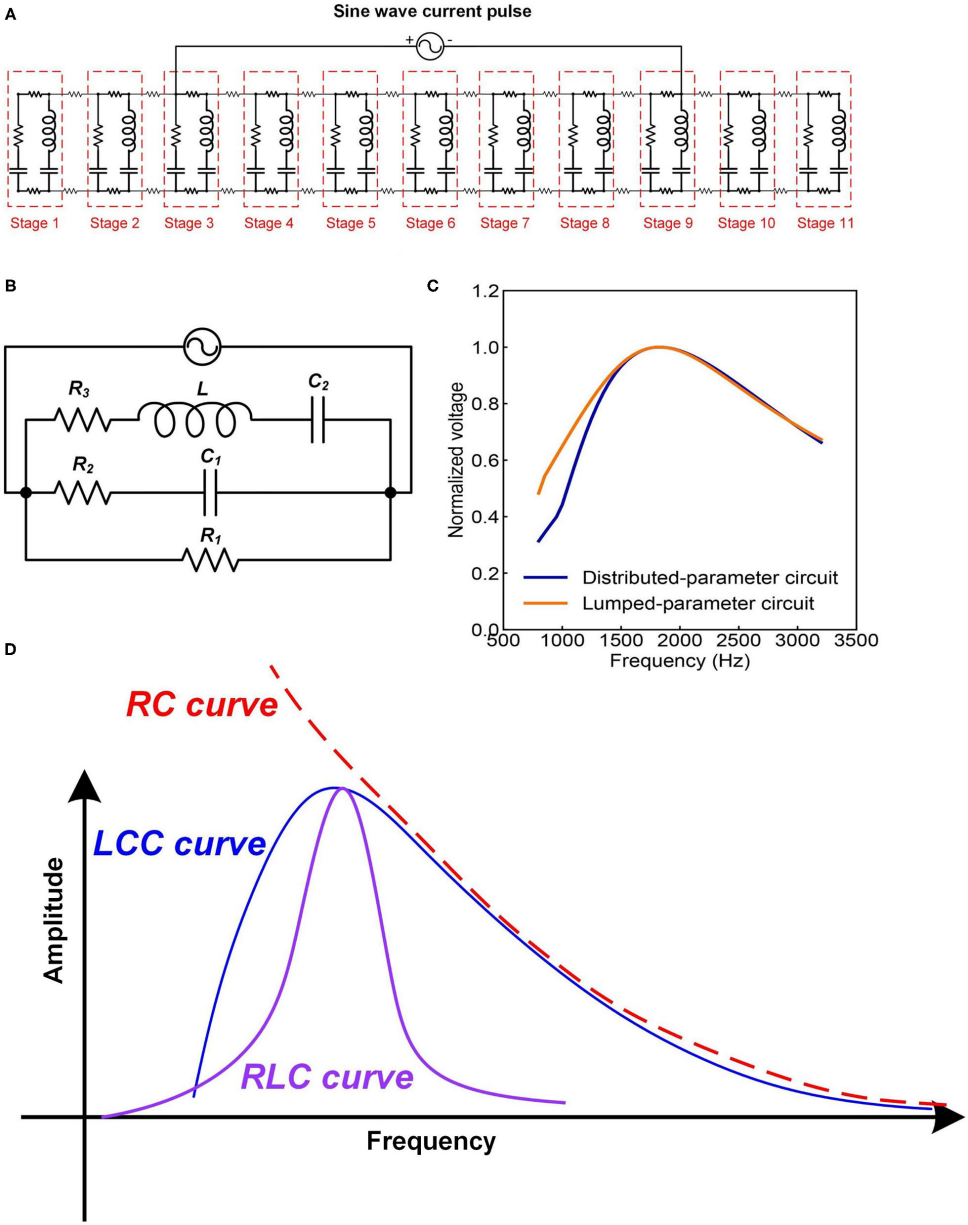
The frequency response of neural circuits. **(A)** The distributed-parameter circuit used in simulation; **(B)** The lumped-parameter circuit used in simulation; **(C)** The frequency response curves of the two circuits; **(D)** A comparison of the frequency response curves of LCC, RLC, and RC circuits.

The most straightforward validation of a circuit with resonance frequency is the characterization of its frequency response. We can analyze the frequency response of this LCC circuit by simulation. Since the resonance frequency of neurons is in the range of kHz or even lower, a resonance frequency of about 1,700 Hz is set. Both the distributed-parameter circuit, which is the cascade, and the lumped-parameter circuit used in the simulation are shown in Figures 7A,B. By rescaling the circuit parameters, we can make the frequency response curves of the two circuits almost overlapped with each other, shown in Figure 7C.

As seen, the frequency response curve of this LCC circuit is asymmetric, which is different from that of a standard RLC circuit, which is symmetric. A brief comparison of the curve shapes of RLC and LCC circuits is shown in Figure 7D. The LCC circuit behaves more like an RC circuit at the high-frequency range. A circuit analysis can also achieve this conclusion. For the LC branch, the impedance of the inductor is *jωL*, which approaches infinite by increasing the frequency. So the LC branch can be simplified as an open-circuit at the high-frequency range. Then the whole cascade is simplified as an RC circuit at the high-frequency range. So we can predict that the frequency response curve of all kinds of neurons should follow the same shape as the LCC curve shown in Figure 7D, which has an asymmetric shape and gets close to an RC circuit at the high-frequency range. The review by Yosef Yarom about the intrinsic frequency response of neurons shows the frequency response curve of neurons (Hutcheon and Yarom, 2000), which is almost the same as the LCC curve in Figure 7D. In that paper, it clearly emphasized that this curve overlaps with an RC circuit at the high-frequency range (P8 in **Figure 14**).

#### The Validation of Stimulus Artifacts Fitting

Another more accurate validation is reproducing the voltage response of the neuron. When a current pulse is applied onto the neuron, there is a voltage generated, which is usually called stimulus artifacts in experiments of the neural signal recording. Since the neuron is not purely resistive, the applied current pulse and measured voltage pulse will never be the same waveform. If the equivalent neural circuit follows the LCC form, we can reproduce the voltage waveform by an LCC circuit. Two groups of experiments were conducted in this study. One is Electroneurogram (ENG) recoding on the sciatic nerve elicited by stimulating the motor cortex. Another is Electromyogram (EMG) recording on the external urethral sphincter (EUS) by stimulating the pelvic nerve. The experimental details can be found in Supplementary S1, S2. The lumped-parameter circuit in Figure 7B is used in modeling.

**Figure 8:**
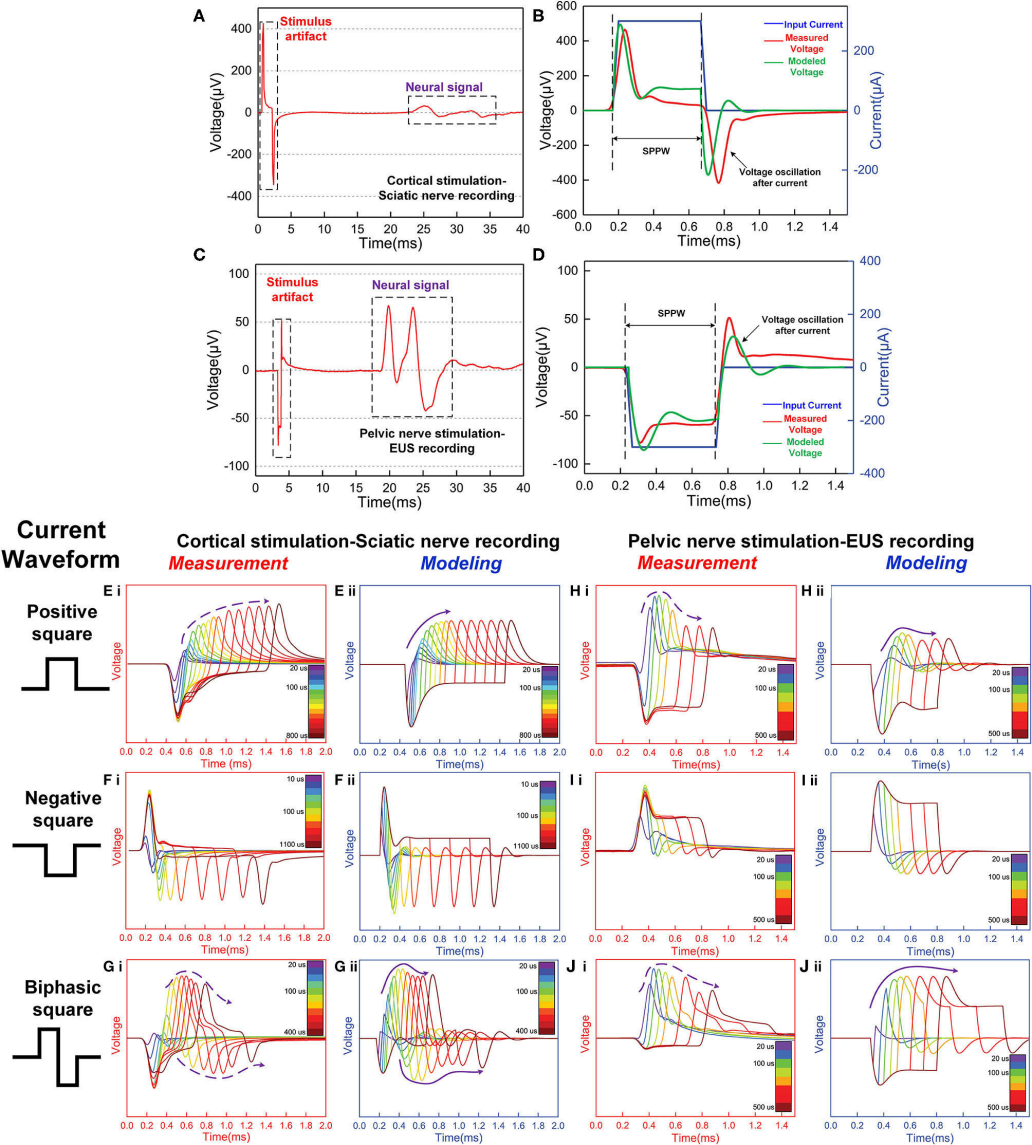
The stimulus artifacts in signal recording in the sciatic nerve and EUS from electrical stimulation of the cortex and pelvic nerve, respectively. These signals can be predicted by the voltage response of an LCC circuit. **(A)** Cortical stimulation elicits conduction in the sciatic nerve. The stimulus artifact generated by the stimulation of cortical neurons is analyzed in **(E–G)**. **(B)** The stimulus artifact in detail: the recorded stimulus artifact (red curve), the applied current (blue curve), and the voltage response of the parallel RLC circuit by modeling (green curve); **(C)** An EMG signal recorded from the external urethral sphincter (EUS) as a result of the pelvic nerve stimulation. The stimulus artifact generated by the nerve stimulation is analyzed in **(H–J)**. **(D)** The stimulus artifact details: the recorded stimulus artifact (red curve), the applied current (blue curve), and the voltage response of the parallel RLC circuit (the green curve); **(E–I)**: Experimental measurement and modeling (notation -i and -ii, respectively) of the stimulus artifact from the peripheral nerve **(E–G)** and pelvic nerve **(H–J)** with different current waveforms and single-phase pulse width (SPPW). **(E–G)** Cortical neuron stimulation -sciatic nerve recording and modeling results: **(E)** positive monophasic square wave, **(F)** negative monophasic square wave, and **(G)** positive-first biphasic square wave; **(H–J)** Pelvic nerve stimulation -EUS recording and modeling results: **(H)** positive monophasic square wave, **(I)** negative monophasic square wave and **(J)** positive-first biphasic square wave; (i) left figures refer to the measured data, (ii) right figures refer to the modeling results. The modeling results match well with the measurement data, validating the LCC circuit used in this study.

Two groups of signals were observed in the signal recording, which is the stimulus artifact and the neural signal (Figures 8A,C). These stimulus artifacts are the voltage response to be fitted by modeling. Figures 8B,D show the zoomed-in stimulus artifacts (red curve) overlapped with input currents (blue curve) and modeling results (green curve). The modeling results mimic the measured voltage oscillation, within and after the current pulses. The modeling parameters in Figures 8B,D can be found in Table 1(f,h). We extend the validation to different input current waveforms and pulse widths (Figures 8E–J). These modeling results [all (ii) figures] duplicate the general voltage waveforms, the voltage oscillation, and, most notably, the resonance effect (indicated by the solid and dashed purple arrows in measurement and modeling results, respectively) in all (i) figures. The modeling parameters can be found in Table 1(e–j).

**Table 1:** Model parameters.

**No**.	***R*_1_(Ω)**	***R*_2_(Ω)**	***R*_3_(Ω)**	***C*1(nF)**	***C*2(nF)**	***L*(H)**
(e)	2,000	1,350	500	10	1,000	0.1464
(f)	3,701	350	500	10	1,000	0.1464
(g)	9,000	1,350	500	10	1,000	0.2326
(h)	80,000	300	1,700	18	1,000	0.1086
(i)	2,656	1,800	800	18	1,000	0.0813
(j)	2,656	1,800	800	18	1,000	0.0813

This is not the first study to record and fit the stimulus artifact (P9 in **Figure 14**). A paper published in 1961 (Araki et al., 1961) reported that a voltage oscillation is recorded on the motorneuron when a square wave current was applied, indicating that the equivalent circuit of the neuron should follow an RLC form. Meanwhile, as pointed out in the above section, this LCC circuit is valid for all kinds of cell membranes, even for plant cells. A paper published in 1984, which is about the pseudo-inductive behavior of the membrane potential of *Chara corallina* under galvanostatic conditions, recorded voltage oscillation generated by a square wave current the same as the data in this study (Homblé and Jenard, 1984). In that paper, it clearly emphasized that this inductive phenomenon is not directly associated with the variant impedance of the ion channels, which is proposed in the H-H model. The original statement is quoted here:

*In Chara corallina, the action potential always fires after the peak of oscillation and we have observed that an overshoot is always present when the cells are refractory. This suggests to us that in plant cells the oscillation is not directly associated with the excitability property*.

Another frequently adopted method to characterize the circuit is by using the Nyquist plot. Since the LCC circuit proposed in this study should prevail for all kinds of neurons, the Nyquist plot reported in Cole's study (Cole, 1941), which characterizes the giant squid axon, should also be achieved by the LCC circuit. In Supplementary S5, a comparison of the Nyquist plots of the RLC circuit proposed by Cole and the LCC circuit proposed by our theory is provided, showing that the LCC circuit can generate the same Nyquist plot as the RLC circuit with proper circuit parameters.

### A New Explanation of Anode Break Excitation (ABE)

Anode break excitation (ABE) is an electrophysiological phenomenon whereby a neuron fires action potentials in response to the termination of a hyperpolarizing current (Huxley, 1963). In other words, when a positive current is applied, there is a chance to activate an action potential at the end of the current pulse. Since only negative voltage can activate the ion channel, this ABE is an unusual phenomenon. Conventionally, ABE is explained by the property of ion channels in the H-H model (Huxley, 1963). Moreover, a similar phenomenon also happens in the stimulation of cardiac tissue (Ranjan et al., 1998). Since cardiac tissue is a non-neural, so a different explanation, called a virtual-cathode hypothesis, is proposed to account for ABE (Wikswo and Abbas, 1995).

In our theory, the ABE in both neural and non-neural tissue has a simple explanation (P10 in **Figure 14**). The key to explain ABE is the mechanism of generating a negative voltage at the end of the positive current. Since the conventional neural theory is based on an RC circuit, it is impossible to generate a negative voltage by applying a positive current. However, if an inductor is involved in the circuit, it is almost inevitable to have a negative voltage overshoot at the end of the positive current, which is exactly the case in Figure 7B. This negative voltage overshoot only happens after the endpoint of the positive current to activate action potentials, which is precisely the observation of ABE. We can further predict that this ABE is a common phenomenon for all kinds of excitable membranes since all cell membranes share the same LCC circuit.

## Chapter 4. How Neurons Are Affected by the Magnetic Field

Since the myelin is treated as a coil inductor, all magnetism related phenomena in neurons shall be theoretically derived from our theory. In particular, these phenomena are observed in magnetic nerve stimulations and magnetic resonance imaging (MRI) of neurons.

### Magnetic Nerve Stimulations

The myelin sheath, acting as a coil inductor, can directly induce electric current by applying an external magnetic field. This induced electric current is coupled onto the node of Ranvier and further activate action potentials. This is the mechanism of magnetic nerve stimulations. Thus, all unique phenomena observed in magnetic nerve stimulation can be simply derived.

#### In Peripheral Nervous Systems, the Magnetic Nerve Stimulation Is Not Determined by the Amplitude, but by the Gradient of the Magnetic Field

According to our theory, a special prediction can be obtained in magnetic nerve stimulations: it is the gradient rather than the amplitude of the magnetic field to affect the magnetic nerve stimulations (P11 in **Figure 14**). The opposite spiraling phenomenon induces it.

**Figure 9:**
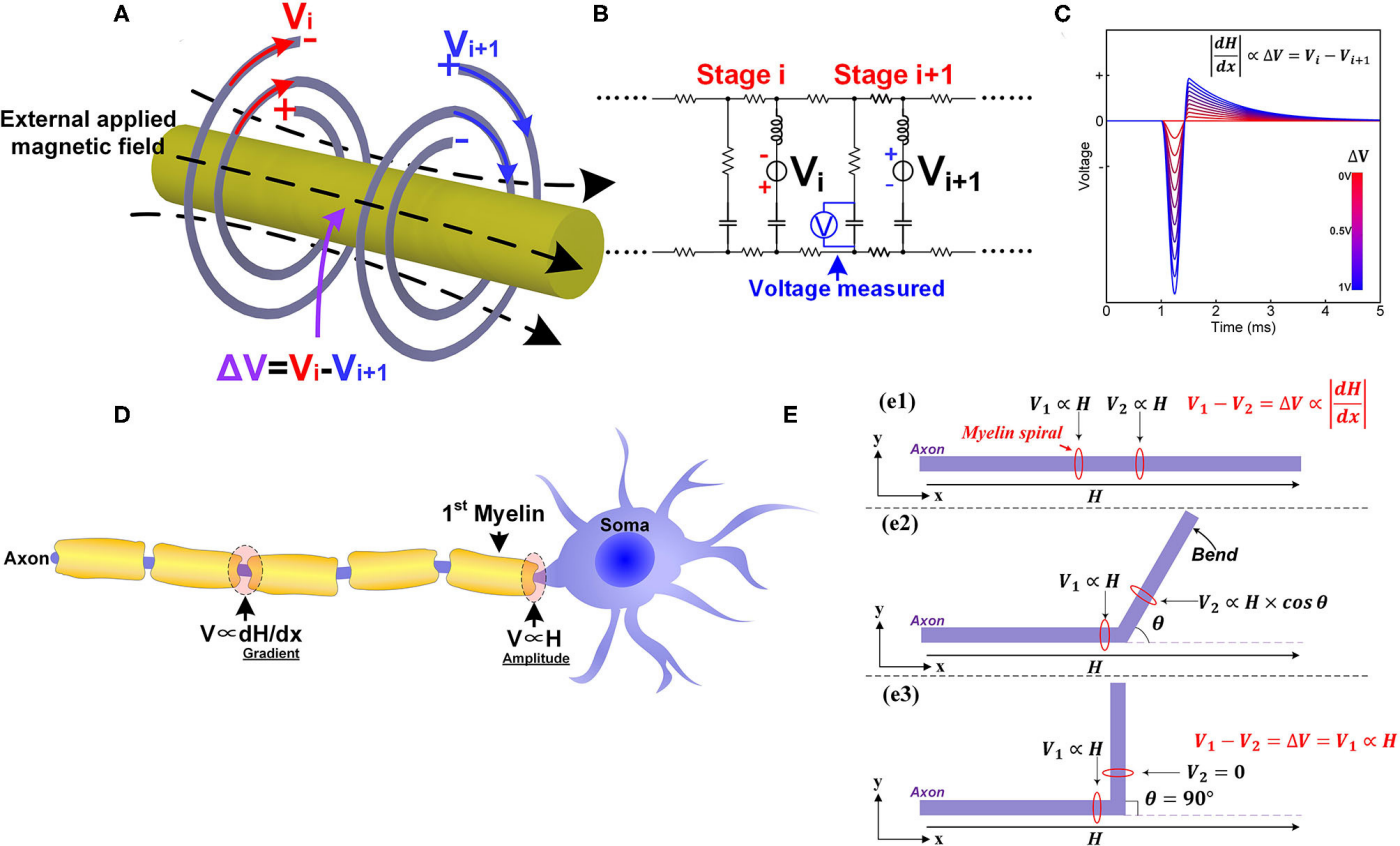
The phenomena in magnetic nerve stimulations. **(A)** The induced potentials on adjacent myelin sheaths are of opposite polarities; **(B)** The circuit used for simulation; **(C)** The simulation results show that the voltage on the node of Ranvier is proportional to the difference of the two voltage sources; **(D)** The different scenarios for magnetic nerve stimulations in peripheral nervous systems and central nervous systems; **(E)** The bending angle of the axon affects the magnetic nerve stimulations.

As coil inductors, their opposite spiraling directions result in opposite polarities of the induced potentials by applying an external magnetic field, as shown in Figure 9A (V_i_ is negative outside and positive inside, V_i+1_ is positive outside and negative inside). These two potentials cancel with each other and only the resulting differential potential (Δ*V* = *V*_*i*_ − *V*_*i*+1_) can be coupled onto the node of Ranvier. Since the amplitude of the induced potential is proportional to the amplitude of the magnetic field, the differential potential, Δ*V*, is proportional to the amplitude difference of the magnetic field upon these two myelin sheaths. This amplitude difference is the spatial gradient of the magnetic field. This is why the spatial gradient can determine magnetic nerve stimulations. Moreover, we can obtain another two deductions:

- Only the component along the axon is capable of stimulating the nerve.
- The magnetic nerve stimulation shares the same nature as electrical nerve stimulation. The myelin sheath provides the electric field by electromagnetic induction.

We can also make this prediction more visible and quantitative by circuit simulation. The circuit follows the same LCC cascade form, as shown in Figure 9B. In this scenario, the two voltage sources with the opposite polarities are connected in series with the two inductors to mimic the electrical potentials provided by myelin sheaths. A voltage meter is connected in parallel with the central capacitor to measure the resulting voltage on the node of Ranvier between to myelin sheaths. The modeling results in Figure 9C shows that the voltage on the node of Ranvier is proportional to the amplitude difference of the two voltage sources.

This gradient effect in magnetic nerve stimulation is a well-known phenomenon reported in lots of studies. An unequivocal statement in one study is quoted here (Irnich, 1994):

*It is the amplitude of the gradient field that is responsible for stimulation and not* $\frac{dB}{dt}$.

It is easy to obtain more deductions, as follow:

- Unmyelinated nerves are theoretically impossible to be stimulated by the magnetic field (P12 in **Figure 14**). They do not have the coil structure to convert the magnetic field to electric potentials. This is also a well-known phenomenon, quoted here (Wang et al., 2018):
  *The activation thresholds of unmyelinated axons obtained with either cable equation are very high and beyond the output capabilities of conventional magnetic stimulators*.
- Compared with the peripheral nervous systems, the position of soma in the central nervous systems will be much easier to be stimulated by the magnetic field (P13 in **Figure 14**). In the scenario of the peripheral nervous system, the induced voltage on the two myelin sheaths will cancel with each other. However, the induced voltage on the first myelin adjacent to the soma will not be canceled by another myelin, shown in Figure 9D. Therefore, the resultant voltage on the node of Ranvier in the peripheral nervous systems will be much lower than that on the first myelin adjacent to the soma. So the position of soma is stimulated by the amplitude of the magnetic field. In contrast, the node of Ranvier in peripheral nervous systems is stimulated by the gradient of the magnetic field. As an experimental observation, the position of soma will be much easier to be stimulated by the magnetic field, which has been validated by previous studies. The original statement is quoted here (Pashut et al., 2011):
  *The largest impact on peripheral neurons was found at the location along the axon experiencing the largest gradient of the induced electric field. However, in CNS neurons, TMS was found to directly depolarize the soma, leading to initiation of an action potential (A.P.) in the initial segment of the axon*.
- In transcranial magnetic stimulation (TMS), the stimulations always happen in the white matter of the cortex. In particular, stimulations will occur at the position of the first myelin sheath, which is at the interface of gray matter (soma) and white matter (myelinated axon) (P14 in **Figure 14**). The original statement in the previous study is quoted here (Seo et al., 2016):
  *The action potentials were initiated at the axons crossing the boundary between gray matter and white matter*.

#### How the Bending Angle of the Axon Affects the Magnetic Nerve Stimulation

According to our theory, the angle between the axon and the magnetic field is also a parameter affecting the result of magnetic nerve stimulations (P15 in **Figure 14**). This is because that the myelin as a coil inductor can only sense the component of magnetic flux perpendicular to the cross-sectional area, which is determined by the intersection angle between the axon the magnetic field. As a deduction, bending the axon can reduce the threshold current required for magnetic nerve stimulations. In particular, a 90-degree bending can achieve a stimulation at the bending point with a minimum threshold current. The explanation for this phenomenon is shown in Figure 9E.

When the axon is straight (Figure 9e1), the inductive potentials of the two adjacent myelin sheaths are denoted as *V*_1_ and *V*_2_. As explained above, the potential difference is proportional to the gradient of the magnetic field:

$$\begin{array}{l} V_{1}-V_{2}=\Delta V\propto |\frac{dH}{dx}| \end{array}$$

The direction along the axon is set as *x* axis.

When a part of the axon is bent with an angle θ as shown in Figure 9e2, the inductive potential *V*_2_ change as:

$$\begin{array}{l} V_{2}\propto H\times \cos\theta \end{array}$$

Generally, *V*_2_ will decrease since cos θ ≤ 1, and *V* will increase, lowering the threshold current required for magnetic nerve stimulation. When the bending angle θ = 90 (Figure 9e3), *V*_2_ = 0, and the theoretical maximum value, Δ*V* = *V*_1_, is achieved, minimizing the current required for magnetic stimulation.

This deduction is the observation in the study of magnetic nerve stimulation (Maccabee et al., 1993). The original statement is quoted here:

*Increasing the angle of the bend from 0 degree to more than 90 deg graded the decrease in the threshold*.

### The Experimental Observation in MRI

**Figure 10:**
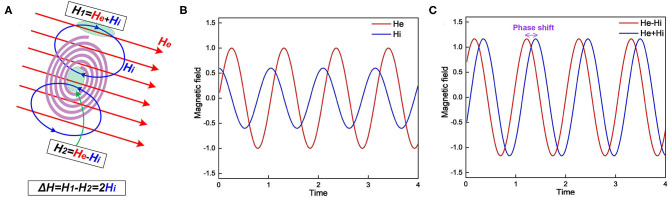
**(A)** The myelin spiral increases the spatial gradient and phase shift of the external magnetic field; **(B)** The external magnetic field He and induced magnetic field Hi; **(C)** The magnetic field inside the spiral, He–Hi, and outside the spiral, He+Hi.

MRI is widely applied for neural imaging. In our theory, the myelin is a significant component in the neuron to interact with the magnetic field, so the unique phenomena observed in MRI should also be deduced. The detailed analysis is shown in Figure 10.

Lenz's law says the direction of the current induced in a conductor by a changing magnetic field is such that the magnetic field created by the induced current opposes the initial changing magnetic field. In the scenario shown in Figure 10A, when the external magnetic field is increasing, the induced magnetic field inside the spiral is in the opposite direction. However, the magnetic field lines is a circle. So the induced magnetic field outside the spiral shares the same direction as the external field. Consider the external magnetic field is *H*_*e*_ and the induced magnetic field is *H*_*i*_. So the total magnetic field strength inside the spiral is *H*_*e*_ − *H*_*i*_ while outside the spiral is *H*_*e*_ + *H*_*i*_. As seen, the existence of the myelin spiral induces a difference of the magnetic field as 2*H*_*i*_. So the myelin will increase the spatial gradient of the magnetic field. For the amplitude-based MRI, myelin can increase the image contrast. Meanwhile, myelin will also affect the phase shift of the magnetic field, explained as follow:

Consider the external applied magnetic field is:

$$\begin{array}{l} H_{e}=A\times \sin\left(\omega t\right) \end{array}$$

Here the physical unit is neglected. Then the induced magnetic *H*_*i*_ is proportional to the derivative of *H*_*e*_:

$$\begin{array}{l} H_{i}=B\times \cos\left(\omega t\right) \end{array}$$

Then the magnetic inside the spiral is:

$$\begin{array}{l} H_{e}-H_{i}=A\times \sin\left(\omega t\right)-B\times \cos\left(\omega t\right) \end{array}$$

The magnetic field outside the spiral is:

$$\begin{array}{l} H_{e}+H_{i}=A\times \sin\left(\omega t\right)+B\times \cos\left(\omega t\right) \end{array}$$

Figure 10B depicts the illustrative curves of *H*_*e*_ and *H*_*i*_. Figure 10C depicts the curves of *H*_*e*_ − *H*_*i*_ and *H*_*e*_ + *H*_*i*_. As seen, these two curves have a phase shift induced by *H*_*i*_. As a result, in the phase-based MRI, the myelin also can increase the image contrast. Then we can obtain deductions as follow:

- Myelin will enhance the image contrast in MRI (P16 in **Figure 14**). The demyelination process will cause a reduction of image contrast in MRI. This is a well-validated observation in previous studies, quoted here:
  *In dysmyelinated shiverer mice, phase imaging correlated strongly with myelin staining, showing reduced contrast. (Lodygensky et al.*, *2012**)*
  *Myelin was proposed as one of the main contributors to M.R. signal phase in white matter, and it was shown that demyelination leads to a loss of phase contrast between white matter (W.M.) and gray matter (G.M.). (Yablonskiy et al.*, *2012**)*
- Since the myelin spiral can only sense the component of the magnetic field, which is perpendicular to its cross-sectional area, the magnetic susceptibility of the myelin sheath is anisotropic (P17 in **Figure 14**). This is also a well-documented phenomenon, quoted here (Xu et al., 2018):
  *There is recent evidence that myelin exhibits susceptibility anisotropy, where the magnetic susceptibility depends on the orientation of the phospholipids in myelin with respect to the magnetic field (Lee et al.*, *2010**; Liu*, *2010**; Li et al.*, *2012**; Wharton and Bowtell*, *2012**; Sati et al.*, *2013**; Sukstanskii and Yablonskiy*, *2014**)*.

## Chapter 5. Theoretical Conjectures About the Biological Functions of Myelin in Nervous Systems

In this chapter, theoretical conjectures of the biological functions of myelin in nervous systems are proposed. These conjectures currently are not validated but can be obtained by further derivation of the theory in this study.

### The Inductive Function of Myelin in Peripheral Nervous Systems

The function of the axon in the peripheral nervous systems can be simplified as a cable for data transmission. Faster transmission speed is the primary purpose of its configuration. In conventional theory, the primary function of the myelin sheath is an insulating layer to inhibit the ionic current on the internodes. So the neural signal can leap from one node of Ranvier to another and thus, enhance the neural signal speed. However, if the myelin is to provide the inductance in the neural circuit, it can improve the signal speed by reducing the spatial decay of neural signals. We will make a detailed circuit analysis from two aspects.

#### Aspect 1: The Inductance Can Maximize the Impedance of Each Stage

**Figure 11:**
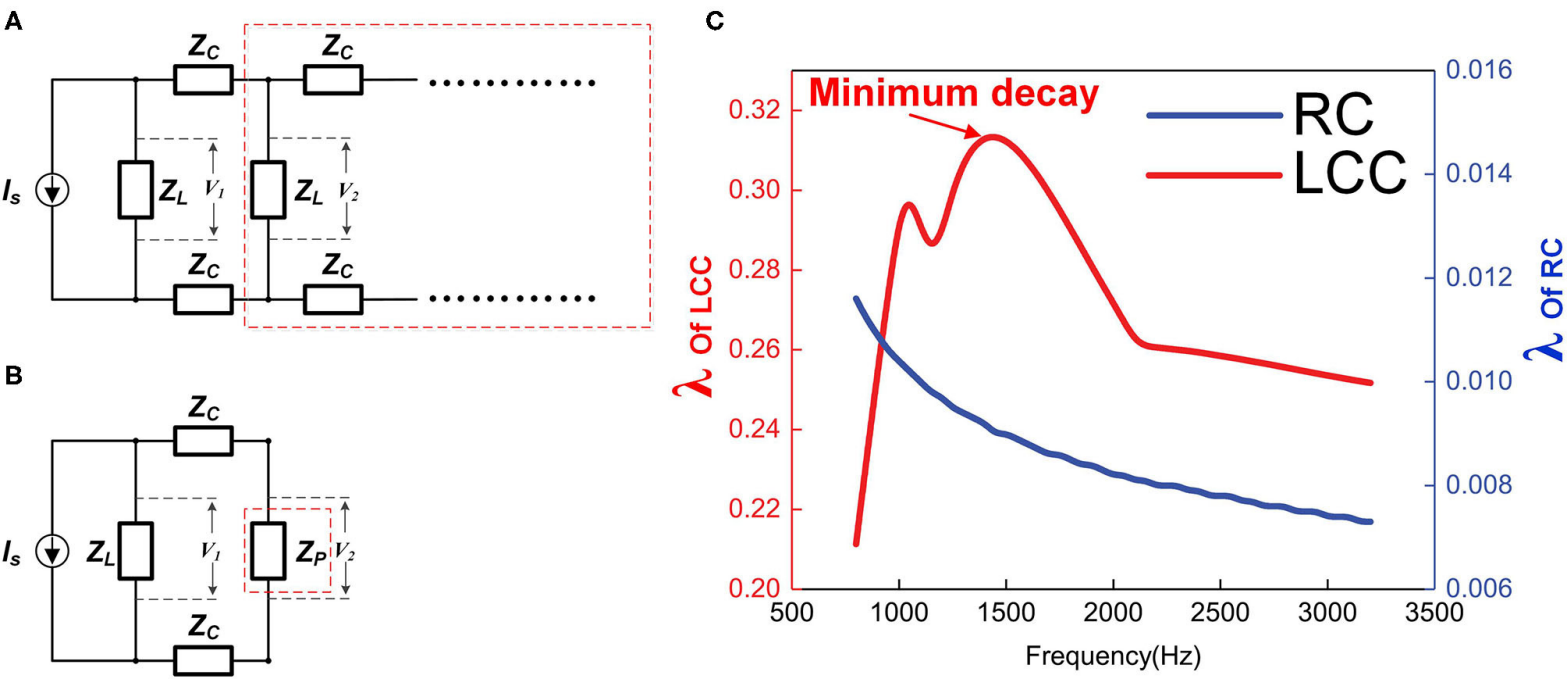
The impedance of each stage affects the signal decay. **(A)** Simplified circuit cascade, **Z**_**L**_ represents the impedance of each stage, which can be RC or L.C. C; **(B)** The circuit cascade in the red dash box in **(A)** is considered as block in the red dash box in **(B)** with impedance of **Z**_**P**_; **(C)** The simulation of the transmission efficiency **λ** of RC and LCC cascades as functions of signal frequency.

The equivalent circuit of an axon can be modeled as a circuit cascade shown in Figure 11A. *Z*_*L*_ represent the impedance of each stage. In the conventional neural circuit, *Z*_*L*_ is the impedance of an RC circuit. In our theory, *Z*_*L*_ is the impedance of an LCC circuit. A current source *I*_*S*_ is connected with the first stage and the voltage amplitude of *nth* stage is denoted as *V*_*n*_. Then define the transmission efficiency λ as:

$$\begin{array}{l} \frac{V_{n+1}}{V_{n}}=\lambda \end{array}$$

Here we will investigate how the λ changed with the impedance *Z*_*L*_.

The total impedance of the whole cascade will converge to a fixed value, denoted as *Z*_*P*_. Since this is a cascade with an infinite number of stages, an extra stage connected to this cascade will not affect the total impedance as shown in Figure 11B, then the equation for *Z*_*P*_ is:

$$\begin{array}{l} Z_{L}//\left({2\times Z}_{C}+Z_{P}\right)=Z_{P} \end{array}$$

Solve this equation:

$$\begin{array}{l} Z_{P}=\sqrt{{Z_{C}}^{2}+2\times Z_{C}\times Z_{L}}-Z_{C} \end{array}$$

Then

$$\begin{array}{l} \lambda =\frac{V_{2}}{V_{1}}=\frac{Z_{P}}{Z_{P}+{2\times Z}_{C}}=\frac{\sqrt{{Z_{C}}^{2}+2\times Z_{C}\times Z_{L}}-Z_{C}}{\sqrt{{Z_{C}}^{2}+2\times Z_{C}\times Z_{L}}+Z_{C}} \end{array}$$

Set $\frac{Z_{L}}{Z_{C}}=\alpha$; then

$$\begin{array}{l} \lambda =\frac{\sqrt{1+2\alpha }-1}{\sqrt{1+2\alpha }+1}=1-\frac{2}{\sqrt{1+2\alpha }+1} \end{array}$$

In this equation, λ increases monotonically with α, and *Z*_*C*_ is a constant value here, so λ increases monotonically with *Z*_*L*_. In other words, a higher load, *Z*_*L*_, results in a higher λ, which means a lower signal decay.

This circuit analysis shows that in this cascade circuit, a higher impedance of each stage can achieve a lower signal decay. Since the actual circuit of each stage is an LCC circuit (RC circuit in conventional theory), this *Z*_*L*_ is a function of the signal frequency. So the signal decay is also a function of the frequency. The frequency here refers to the pulse width of the signal, not the time interval between signal pulses.

The impedance of an RC circuit decreases monotonically with the frequency, while the impedance of an LCC circuit reaches the maximum at the resonance frequency. Therefore, if the frequency of the signal is the same as the resonance frequency of the LCC circuit, it can have the minimum signal decay on this axon. Qualitative simulation of the transmission efficiency, λ, for both RC cascade and LCC cascade is shown in Figure 11C (RC cascade is the same circuit of LCC cascade by removing inductors). As seen, with the same circuit parameters, the transmission efficiency of the LCC cascade is one order higher than that of the RC cascade (note the two axes on both sides). Apparently, an RC cascade without resonance is not suitable for signal transmission, which is also a common sense in engineering. The first undersea cable based on the ideas of William Thompson, Lord Kelvin, and described as an RC cable by Hermann was a technical and financial disaster. Two years later, a more sophisticated RLC cable based on Maxwell's Equations for a coaxial structure was laid with great success. No RC cable has ever been used in practice since that time. There is the same principle for axon as a cable for signal transmission: the resonance frequency of the axon matches with the frequency of the neural signal to achieve the minimum signal decay to maximize the signal speed (P18 in **Figure 14**).

#### Aspect 2: The Opposite Spiraling Can Introduce a Positive Mutual Inductance to Minimize the Signal Decay

**Figure 12:**
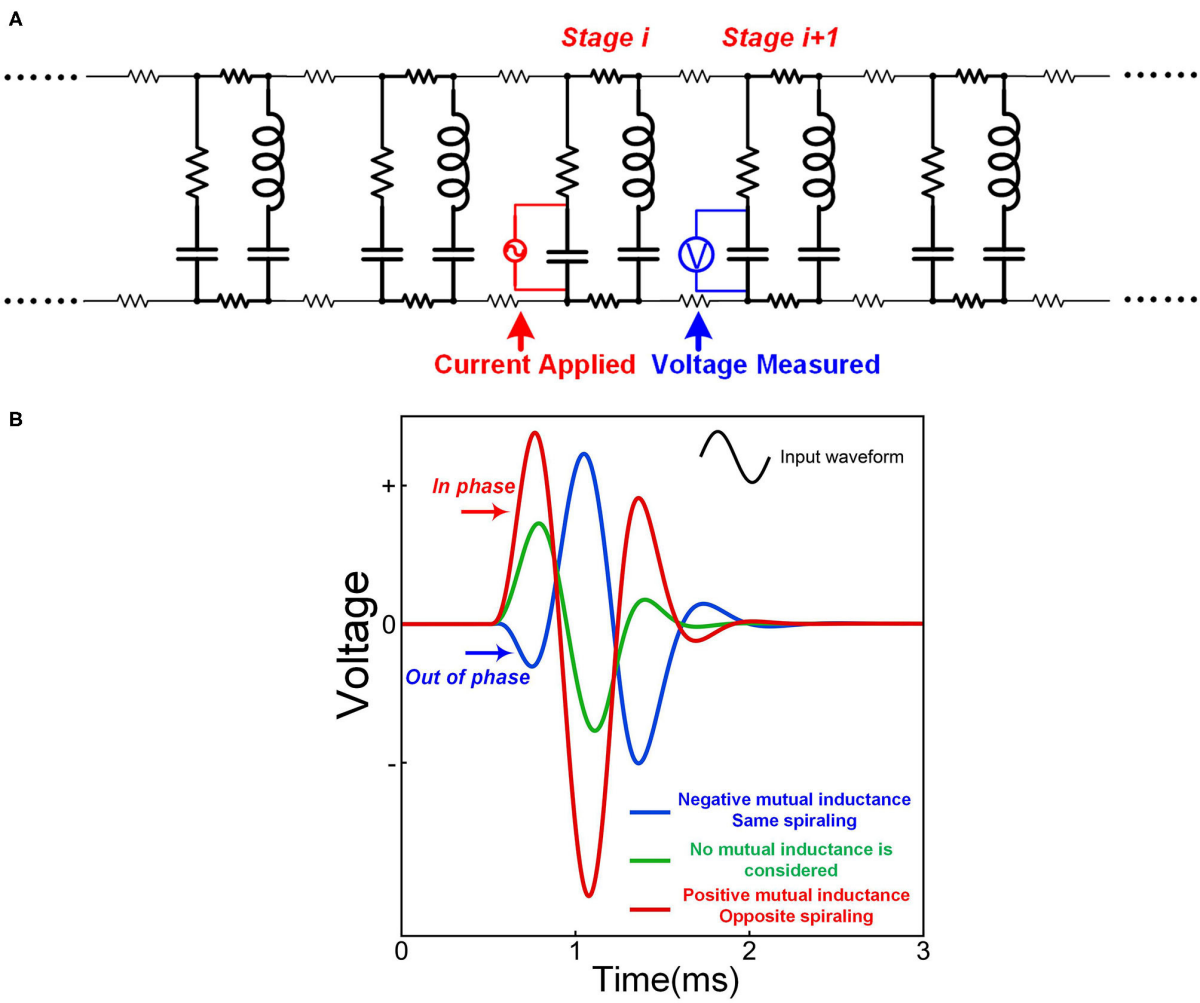
The opposite spiraling can reduce signal decay. **(A)** The circuit for simulation; **(B)** The measured voltage by setting different mutual inductance parameters.

As explained in Figure 2, the opposite spiraling phenomenon can induce the same voltage polarity on the adjacent myelin sheaths. This means a positive mutual inductance in the circuit. Thus, the effect of this opposite spiraling can be modeled by adding the mutual inductance in the circuit shown in Figure 12A. A sinewave pulse is applied to the node of Ranvier of stage *i* to model the generation of an action potential, shown as the applied current in Figure 12A. The voltage waveforms on the node of Ranvier of stage *i* + 1, shown as the measured voltage in Figure 12B, with positive, negative and zero mutual inductances, are compared. The positive mutual inductance achieves the highest amplitude with an in-phase waveform as the current source (positive first). Such an in-phase voltage response enhances the neural signal propagation. This is how the opposite spiraling phenomenon generates a positive influence on the conduction of action potentials (P19 in **Figure 14**).

### The Inductive Function of Myelin in Central Nervous Systems

**Figure 13:**
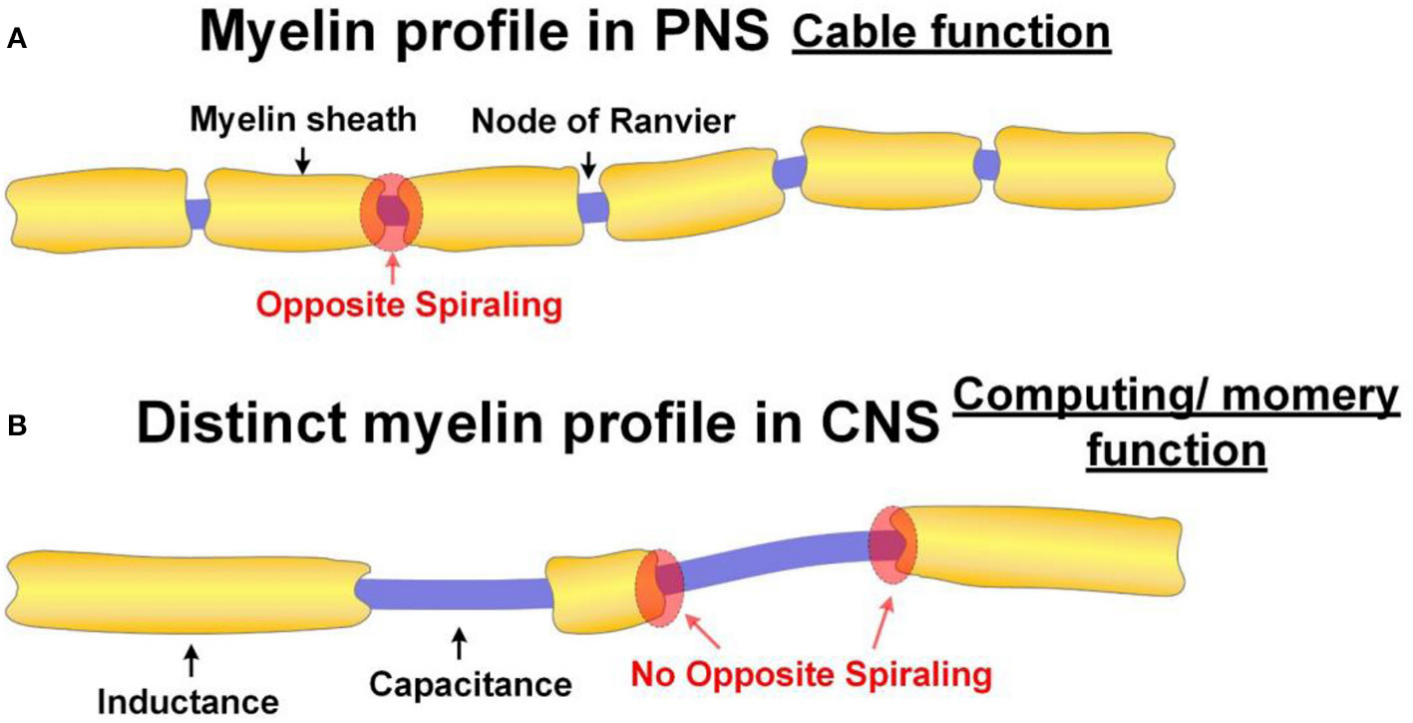
Different myelin profiles in PNS and CNS. **(A)** The normal myelin profile in PNS. The function of the axon with normal myelin profile has the function as a cable; **(B)** The distinct myelin profile in CNS. The axon with this distinct myelin profile has the function of computing or memory.

The peripheral nerves are configured for maximizing the signal transmission speed since their function is to transmit signals. As explained above, the exclusive purpose of the opposite spiraling phenomenon is to achieve a positive mutual inductance to minimize the signal decay. The prerequisite for this mutual inductance is the narrow node of Ranvier. This is why the profile of myelin sheaths always follows the form shown in Figure 13A.

However, central nerves are more than cables. They are also in charge of computing and memory. So the opposite spiraling phenomenon is not necessary for central nerves. By changing the profile of myelin sheaths, for example, leaving a long unmyelinated section can introduce a new property, which is frequency modulation. In particular, the lengths of the internode and Ranvier node can change the parameters of the L and C in the neural circuit and further control the resonance frequency. This may be how neurons in cortex achieve functional differentiation. This is also the actual phenomenon observed in the previous study, quoted here (Tomassy et al., 2014):

*Neurons in the superficial layers displayed the most diversified profiles, including a new pattern where myelinated segments are interspersed with long, unmyelinated tracts*.

An illustrative drawing of this observation is shown in Figure 13B. This phenomenon cannot be explained by the conventional theory, which considers the myelin sheath an insulation layer. So according to this finding, in the paper published in 2014 by R. Douglas Fields, there are statements quoted here (Fields, 2014):

*However, nerve impulses are not transmitted through neuronal axons the way electrons are conducted through a copper wire, and the myelin sheath is far more than an insulator*.

*Tomassy et al. (**2014**) provide a high resolution global view of myelin structure spanning the six layers of mammalian cerebral cortex. The findings are likely to spark new concepts about how information is transmitted and integrated in the brain*.

**Figure 14:**
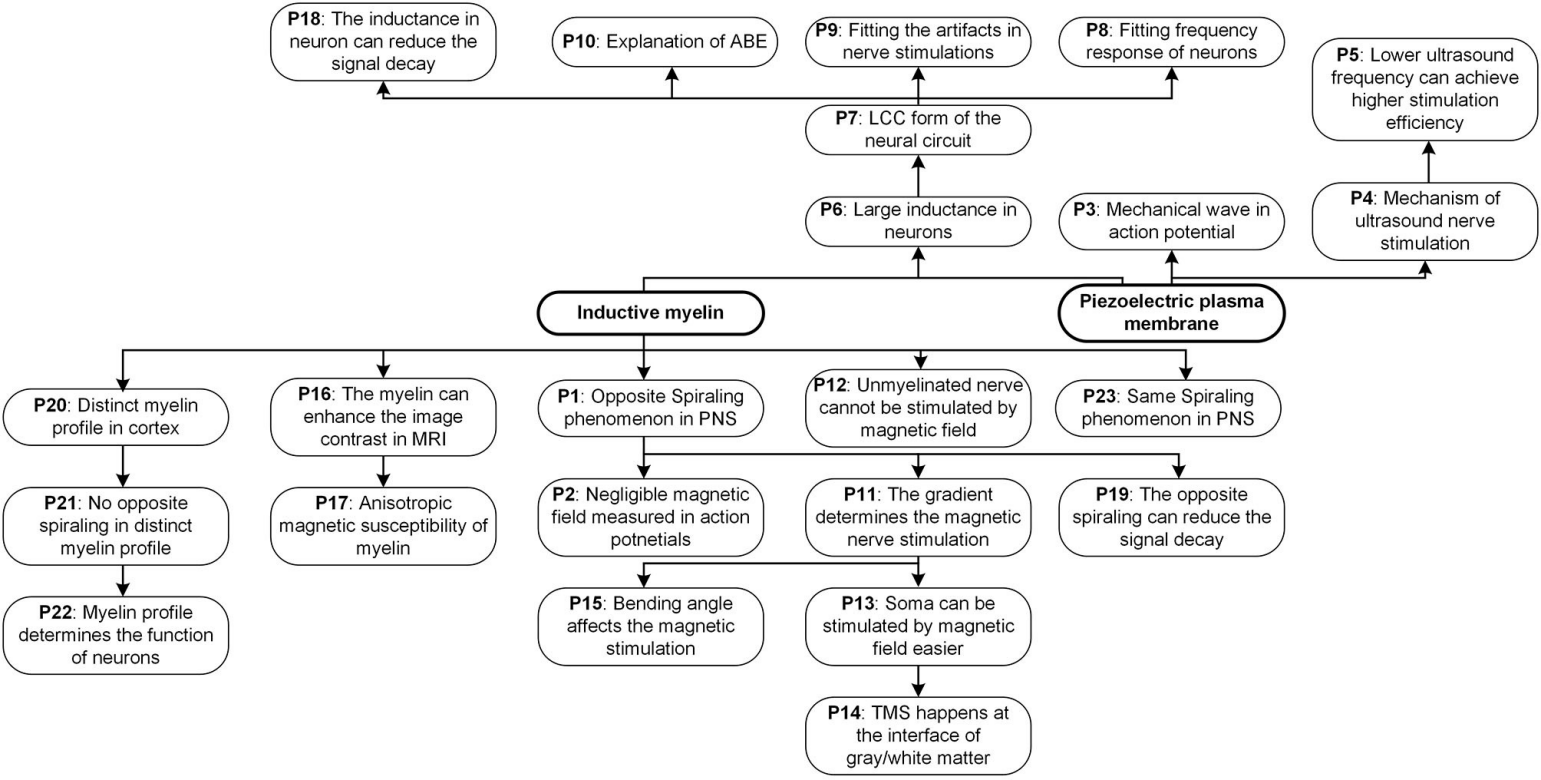
All phenomena and predictions explained in our theory.

Now our theory provides an explanation. This distinct myelin profile is to modulate the resonance frequency of the axon, which is associated with the function of computing or memory (P20 in Figure 14).

Moreover, in the scenario, when there is a long unmyelinated section between two adjacent myelin sheaths, the opposite spiraling phenomenon shall not happen. This is a testable prediction, which can be verified in the future (P21 in Figure 14).

If our theory is correct, there is a simple principle to distinguish the functions of neurons in the cortex. The neurons for signal transmission will have regular myelin profile, as shown in Figure 13A with opposite spiraling. The neurons for computing or memory will have an irregular myelin profile, as shown in Figure 13B (P22 in Figure 14).

## Chapter 6. Summary of All Phenomena and Predictions

All phenomena and predictions explained in our theory are summarized in Figure 14, showing the logic relation between them. This figure can give a more explicit and systematic understanding of the whole theory.

## Conclusion

Two physical origins, which are the coil inductance of myelin and the piezoelectric effect of the cell membrane, are proposed to account for the inductance in neurons. Based on these two hypotheses, a series of observed phenomena, such as the regular spiraling of myelin sheaths and the measured mechanical wave accompany with action potential, are explained. Meanwhile, a new multi-physical perspective of the neural signal is proposed. A modified neural circuit with inductance is developed to reproduce a series of experimental phenomena by modeling. Finally, the biological function of the inductive myelin is summarized.

## Data Availability Statement

The original contributions presented in the study are included in the article/Supplementary Materials, further inquiries can be directed to the corresponding author/s.

## Author Contributions

The theory was developed by HW. The modeling work was carried out by HW, JW, GC, YL, and TW. The major framework of experiment design was carried out by HW and JW. The manuscript was written by HW, JW, and YQ. All authors discussed the experimental results and contributed to the final version of the manuscript.

## Conflict of Interest

The authors declare that the research was conducted in the absence of any commercial or financial relationships that could be construed as a potential conflict of interest.
