# Supplementary material for: A Physical Perspective to the Inductive Function of Myelin—A Missing Piece of Neuroscience
Source: Front Neural Circuits. 2021 Jan 18;14:562005. doi: 10.3389/fncir.2020.562005 (PMC7848263; doi:10.3389/fncir.2020.562005)
Supplement: Supplementary file 1 [file Data_Sheet_1.pdf]

Supplementary Materials for

A physical perspective to the inductive function of myelin - A missing piece of neuroscience

Hao Wang, Jiahui Wang, Guangyi Cai, Yonghong Liu, Yansong Qu and Tianzhun Wu

Correspondence to: hao.wang@siat.ac.cn

This PDF file includes:

S1. Cortical stimulation with sciatic nerve recording test electrode configuration, testing setup, detailed procedure and testing parameters

S2. Pelvic nerve test electrode configuration, testing setup, detailed procedure and testing parameters

S3. Simulation for the inductance of the myelin sheath

S4. Possible experiments proposed for the further validation of this theory

S5 The comparison of the Nyquist plot of RLC circuit proposed by Cole and the LCC circuit proposed in our theory.

S1. Cortical stimulation with sciatic nerve recording test electrode configuration, testing setup, detailed procedure and testing parameters

Animals

Female Sprague-Dawley rats (200-300g) were used in the acute experiments. Rats were housed and cared for in compliance with the guidelines of the National Advisory Committee for Laboratory Animal Research (NACLAR) and were humanely euthanized after the experiment. During the experiment, isoflurane was used to induce general anesthesia (Aerrane®, Baxter Healthcare Corp., USA) prior to injection of ketamine/xylazine (37.5% ketamine, 5% xylazine, 0. 2ml / 100 g Comparative Medicine, NUS). Paw retraction reflex and breathing rate was used to assess the depth of anesthesia, and the core body temperature at 37°C was maintained using a heating pad (Stryker T/pump, Kent Scientific Corp., USA). Rats were then placed in a stereotaxic frame (Kopf instruments, USA). Microsurgical techniques were used to expose the left sciatic nerve, the tibialis anterior and the skull, which was then subjected to a craniotomy to access the right motor cortex.

Stimulation and Recording

A tungsten single shank electrode (0.5MΩ, Microprobe Inc, USA) was implanted into the motor cortex in several locations at a depth of 2.0mm (near the layer V motor cortex pyramidal neurons) until the tibialis anterior was observed to move with a stimulation (100 µA). Once a movement was observed, the location was used for the subsequent experiment.

A thin-filament longitudinal intrafascicular electrode was used in the sciatic nerve to record stimulation artifacts and nerve activity during stimulation. Furthermore, using a string tied to the hind paw of the rat attached to a dynamometer (Hand dynamometer, Vernier, USA with NI-DAQ USB-6008, National instruments, USA), force was recorded simultaneously.

S2. Pelvic nerve test electrode configuration, testing setup, detailed procedure and testing parameters

Animal subject and surgery

Female Sprague-Dawley rats (200-300 g) were used in the acute experiments. The animals were housed in pairs in individually ventilated cages, maintained in a 22-24°C room with a 12 h light–dark cycle, and given ad libitum access to food and water. All procedures were performed in accordance with protocols approved by the Institutional Animal Care and Use Committee of the National University of Singapore. For each experiment, the animal was anesthetized with a mixture (0.2 ml/100 g) of ketamine (37.5 mg/ml) and xylazine (5 mg/ml) intraperitoneally (I.P.) for induction, and a supplementary dose of 0.1 ml/100 g was injected I.P. for maintenance as required. The animal was placed in the supine position, and kept warm with a water-circulating heating pad. Laparotomy was performed, and underlying muscles were cut and adipose and connective tissues were gently teased apart to expose the pelvic nerve branches for electrical stimulation. To record muscle activity from the external urethral sphincter (EUS), the pubic bone overlying the urethra was cut and fat tissues were teased apart.

Pelvic Nerve stimulation and EUS EMG recording

Hook electrodes made from platinum iridium wires (A-M systems, 0.005" bare, 0.008" coated) were implanted onto the pelvic nerve branches unilaterally and silicone elastomer (Kwik-Sil, World Precision Instruments, FL, USA) was used to encase the electrode-nerve interface. A commercial isolated stimulator (A-M systems model 2100, WA, USA) was used to deliver either cathodic, anodic or biphasic rectangular pulse for pelvic nerve stimulation at a repetition frequency of 2 Hz and current amplitude of 70 μA and different pulse widths (20, 60, 100, 150, 200, 300, 400, 500 μs) used for each set of stimulation. A pair of fine stainless steel wires (304, California Fine Wire, CA, USA) with exposed tips was sutured to the top of the exposed urethra beneath the dissected pubic bone to record EUS EMG signals. EMG signals were amplified by using an Intan preamplifier 2216, and acquired at 20 kHz with the Intan RHD2000 data acquisition board (Intan Technologies), with a 50 Hz notch filter. Stimulation pulse markers were sent from the stimulator to the data acquisition board (DAQ) collecting EMG data simultaneously for data synchronization.

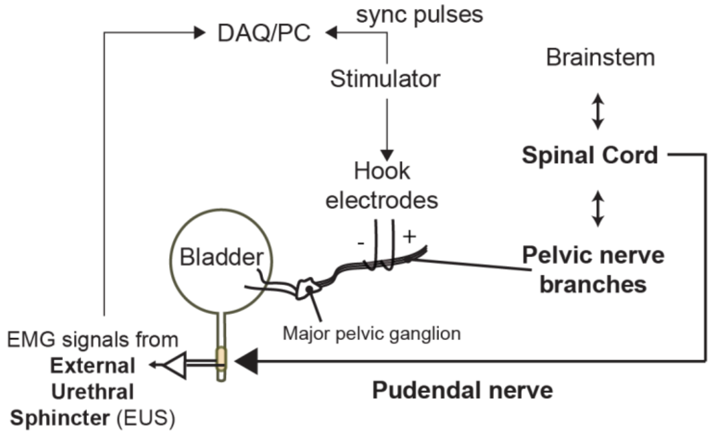

Fig. S2. Pelvic Nerve stimulation and EUS EMG recording

S3. Simulation for the inductance of the myelin sheath

The calculation of the inductance of the myelin is a quite complex task. The inductance of each physical entity is determined by its geometry. Only those coils with some specific shapes, such as a normal coil, have some formulae to calculate their inductance. The myelin sheath is a spiral sheet, which is different from a normal coil. Currently, there is no such mathematical model for its inductance calculation. We can only obtain the inductance value by simulation.

We used COMSOL for this simulation. The 3D model of the myelin sheath is shown in Figure S3.1. All the physical parameters were very close to the actual parameters. The inner diameter of the axon is 1.2 µm. The thickness of each layer is 10 nm and the spacing between the two layers is 4 nm, which is close to the actual biological structure of the myelin sheath. The conductivity is set as copper. Since the conductivity normally will not affect the value of inductance, here we just set it as a conductive metal sheet. There are two variables: the round of spirals and the length. The inner layer of the out layer are connected to a power source, and then a stimulus will be applied. The voltage response on the spiral sheet will be calculated. The voltage response will be fitted with a standard RLC circuit. The parameter of the inductance is extracted as the inductance value of the spiral sheet.

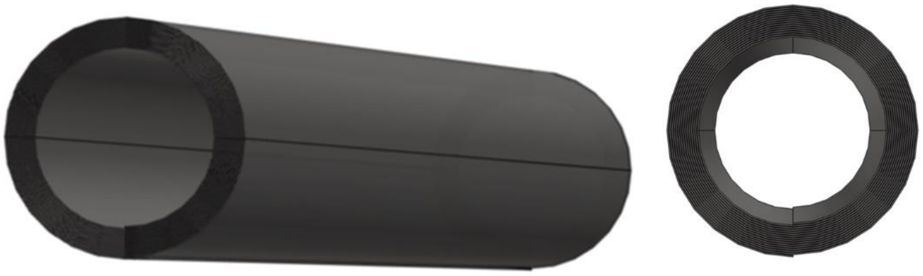

Fig S3.1 The 3D model of the myelin sheath for simulation.

When the length of the myelin sheath is 10nm, the curve of the inductance changing with the number of rounds is shown in Figure S3.2(a). This curve can be fitted with a quadratic function as below:

$$L = 7.12224 \times 10^{-13} \times n^2 + 4.88303 \times 10^{-12} \times n - 3.3974 \times 10^{-12}$$

Here n is the number of rounds. Since in this simulation, the myelin sheath is 10 nm in length, making it similar to a typical coil. In the empirical equation for the inductance calculation, $L \propto n^2$. So the equation fitting the simulation follows the same quadratic format.

Then the inductance changing with the length of the myelin is shown in Figure S3.2(b). As seen, increasing the length of the myelin decreases the value of the inductance. However, the inductance seems to converge at a certain value if further increases the length. Due to the limitation of our computer, we are not able to simulate a longer myelin sheath.

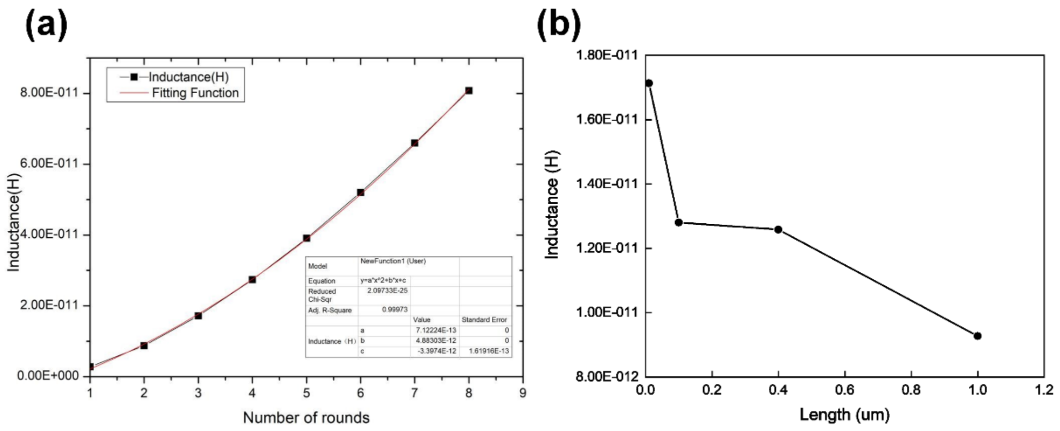

Fig. S3.2 (a) The inductance changes with the number of rounds when the length is 10 nm; (b) The inductance change with the length when the number of rounds is 3.

Based on this simulation, we roughly know the range of the inductance of a myelin sheath. For a normal myelin sheath with 15 rounds, its inductance is about 0.23 nH when the length is 10 nm. The actual length of a myelin sheath may be at 100 um level, which will decrease this value a lot. Then for the myelin sheath in the cortex, whose rounds can be 150, the inductance can be at the range of nH.

S4. Possible experiments proposed for further validation of this theory.

Here we proposed some possible experiments which can further validate the hypotheses in this theory.

1. Measurement of the magnetic field in action potentials of a single myelinated nerve fiber.

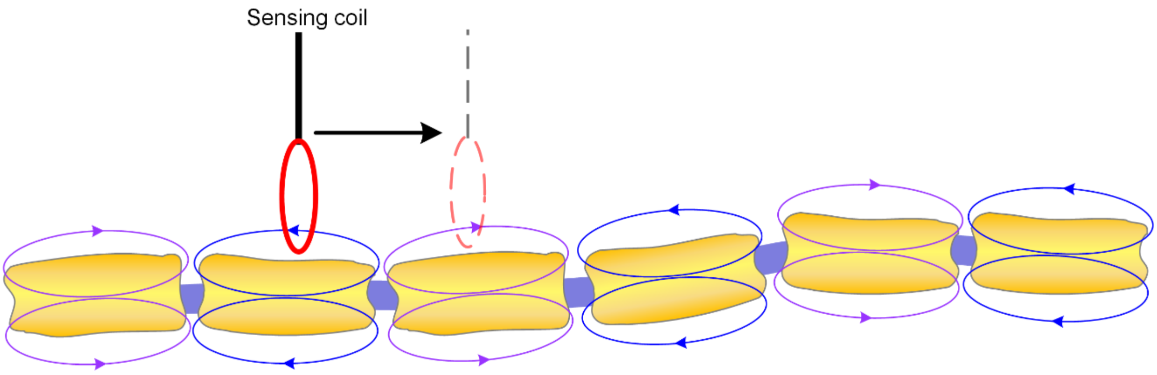

Fig. 4.1 Proposed experiment of magnetic field measurement in action potentials of a single myelinated nerve fiber.

According to our theory, the magnetic field generated by the adjacent myelin sheath should be of opposite directions due to their opposite spiraling directions. If we can prepare a MEMS-based coil to measure the magnetic field at the position of each myelin, the measured current induced by the magnetic field from the myelin when an action potential pass-through should be of opposite polarities. This is a unique phenomenon that cannot be obtained from any theories up to now. This experiment can validate two points:

a. The spiraling of the myelin can generate a magnetic field in the action potential.
b. The opposite spiraling phenomenon plays a role in the propagation of action potentials.

2. In vitro myelination experiment

The opposite spiraling phenomenon indicates that the action potential propagation and activation play an important role in the myelination process. Therefore, during the myelination process, if there is no neural signal generated on that nerve fiber, this opposite spiraling phenomenon should not be observed. Thus, an in vitro myelination experiment can be performed. During the whole myelination process, avoid all electrical disturbance on the nerve fiber. Then after the myelination process is finished, check the spiraling direction of adjacent myelin sheaths in SEM. If this theory is correct, the opposite spiraling phenomenon should not be observed.

3. A statistical observation of the opposite spiraling phenomenon in PNS

In this theory, it is proposed that the major biological function of myelin sheaths in PNS is to provide positive mutual inductance between adjacent myelin sheaths. If this conclusion is correct, this opposite spiraling phenomenon should be observed in all nerve fibers in PNS from all animals. Although the first observation of this opposite spiraling is on the sciatic nerve of mice in 1957, in the past half-century, no one tried to validate it on other nervous

systems of other animals. Since this opposite spiraling phenomenon cannot be obtained by any other theories, a statistical observation of this phenomenon should be the most substantial evidence.

S5. The comparison of the Nyquist plot of RLC circuit proposed by Cole and the LCC circuit proposed in our theory.

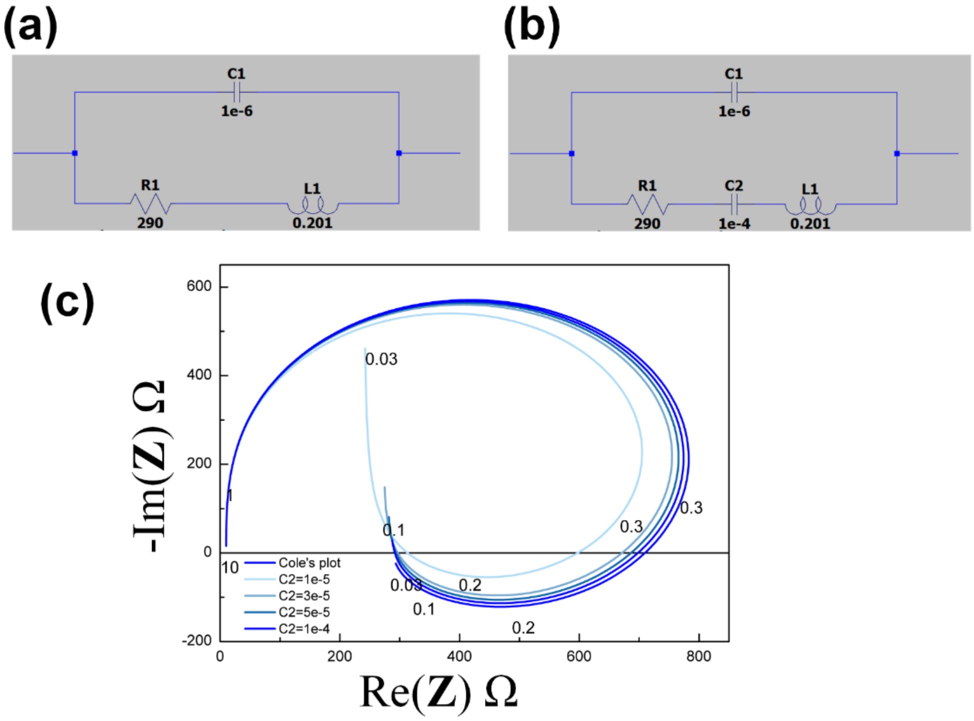

Fig. S5. (a) The RLC circuit proposed by Cole; (b) The LCC circuit proposed by our theory; (c) The Nyquist plot of the RLC and LCC circuit.

Here we compare the Nyquist plot of the RLC circuit proposed by Cole and the LCC circuit proposed by our theory. The detailed circuit parameters of these two circuits are shown in Fig. S5 (a & b). These circuit parameters follow similar values proposed in Cole's study [1].

The results of the Nyquist plot is shown in Fig. S5 (c). As seen, by increasing the value of C2, the LCC circuit curve will gradually get close to the curve of the RLC circuit. As seen, in the LCC circuit, to fit the Nyquist plot of the neuron, C2 should be much larger than C1.

Reference

[1] Cole, K.S., 1941. Rectification and inductance in the squid giant axon. The Journal of general physiology, 25(1), pp.29-51.
